# A New Analysis of the Mechanics of Fixed-Plug Tube Drawing with Application on a Low Carbon Steel Tube

**DOI:** 10.3390/ma19194235

**Published:** 2026-10-06

**Authors:** Miklos Palkovics, Laszlo S. Toth

**Affiliations:** 1 Institute of Physical Metallurgy, Metal Forming and Nanotechnology, University of Miskolc, H-3515 Miskolc, Hungary; miklos.palkovics@student.uni-miskolc.hu; 2 Université de Lorraine, CNRS, Arts et Métiers ParisTech, LEM3, F-57000 Metz, France

**Keywords:** tube drawing, drawing force, stress and strain analysis, finite element simulation, crystallographic texture

## Abstract

Knowledge of the deformations that occur during the plastic forming of metals and the necessary forces is of key importance in engineering. The Fixed-Plug Tube Drawing (FPTD) process permits reductions of the diameter and the wall thickness of a tube to the desired geometry. In this study, a new analysis of the mechanics of the FPTD process is presented. The analysis was inspired by finite element simulations of the process. It has been found that the second stage of the main deformation process within the die can be described by a simple shear deformation mode. Using this principle, the velocity gradient field and the stress state for the drawing force was determined, and a new formula, much simpler than the existing Geleji formula, was obtained. The new modeling was validated by comparing the simulated drawing force to the experimental one, showing slightly better agreement than the finite element simulations or the Geleji formula. The comparison of the measured and VPSC-simulated textures further validated the new analytical approach. It has been found that the textures obtained in FPTD are highly similar to the textures observed in cross-rolling low carbon steel. The similarity, however, was obtained between different pole figures, the reason for which is the difference in strain paths.

## 1. Introduction

In almost all areas of industry, steel tubes have properties that go beyond those of a simple raw material. They have to withstand a wide range of external and internal conditions, such as the fluids and gases that flow through them, external environmental influences, and the mechanical and chemical specifications set by the standards.

A special area of tube manufacturing is cold drawing, where plastic deformation is obtained by a combination of tensile and compressive stresses. Cold drawing can achieve tighter dimensional tolerances [1], better surface quality [2], improved mechanical properties [3], and thinner-walled tubes than hot forming. The simplest method of cold drawing is sinking, which is used to reduce the diameter. However, the internal diameter and wall thickness cannot be controlled, and the wall thickness increases during the process. Accurate control of the inside diameter requires the use of a fixed plug, mandrel or floating plug inserted inside the tube during the drawing process [4]. When using fixed-plug drawing, the plug is fixed to a long support rod, which forms an annular gap with the tool through which the tube is drawn. Mandrel drawing is used mainly for standard sizes and as a so-called pre-drawing process, where the final dimensions are produced by several successive drawing operations with intermediate heat treatment. In flying or floating plug drawing, as the name suggests, the plug floats in the tube and is designed to find a suitable position in the reduction zone of the tool. This type of drawing is typically used for small diameter and longer lengths of tube, as there is no length limitation on the length of the tube. As an innovation in manufacturing technology, the use of shape plug drawing has been driven by market demand for the production of tubes with complex shaped internal grooved surfaces, mainly used in energy applications. The tubes are drawn in several passes, and the final draw uses a special free-rotating shaped plug with multiple spiral grooves to create a spiral grooved pattern on the inner surface of the tube [5].

Several recent studies have investigated different aspects of tube drawing. Among these is the study by Al-Hamdany et al. [6], which investigates how the use of a tilted drawing die affects the wall thickness eccentricity of copper tubes. The objective of the research was to control and reduce unevenness in the wall thicknesses of the tubes. The authors demonstrated that the magnitude and direction of tube eccentricity can be controlled by adjusting the tilt angle of the drawing die. Razali et al. [7] also investigated the potential for reducing the raw material-induced eccentricity and wall thickness defects using a tilted drawing die by means of a finite element simulation model. They applied a so-called multi-body treatment scheme (MBTS) within an implicit elastoplastic framework, in which the internal plug was treated as a deformable body capable of lateral displacement. Their results showed that an appropriately tilted drawing die can substantially reduce tube eccentricity and wall thickness non-uniformity. The publication by Gattmah et al. [8] focused on the precise measurement of residual internal stresses in the tube wall following cold tube drawing. They developed a new, more accurate experimental-analytical measurement method to quantify the residual stresses remaining after the fixed plug drawing process. The precision fixed plug cold drawing of thin-walled, work-hardening CuSn8 alloy tubes was investigated in a publication by Suliga et al. [9]. The authors simulated the stress and strain states, temperature rise, and tool wear using the finite element method (FEM). The most significant finding of the research was that the semi-cone angle of the drawing die strongly influences plug wear, the magnitude of the drawing force, and material failure. The optimum drawing angle reported for the investigated thin-walled tubes was 2*θ* = 22°, at which approximately a 10% reduction in drawing force was obtained. Khani et al. [10] analyzed the development of crystallographic texture during tube drawing in the case of seamless copper tubes drawn with an oblique die, paying particular attention to texture changes between different regions of the tube’s cross-section. In their research, they combined diffraction techniques with electron backscatter diffraction (EBSD) to characterize the changes in crystallographic orientation induced by the drawing process. The results showed that the deformation history associated with tube drawing creates distinct texture characteristics that vary depending on the local deformation conditions within the tube wall.

The solution to questions concerning forming technology can be achieved analytically through several computational methods, which are based on different mechanical models. Such an example is Avitzur’s (1983) [11] solution applied to tube sinking, or its adaptation by Neves et al. [12] to fixed-plug tube drawing, where an analytical upper-bound model was developed in a cylindrical coordinate system under the Tresca flow condition. Several studies apply the energy method under constant yield strength, such as the analytical model developed by Um and Lee [13], which uses a kinematically admissible velocity field together with energy considerations within the Upper Bound method (UB Method). Their analytical solution was developed for tube drawing with a fixed tapered plug and can also be reduced to the tube-sinking case by setting the friction factor between the tube and the plug to zero. The UB Method is suitable for estimating drawing stress and determining optimal tool geometry and process parameters. Rubio et al. [14] also applied the UB Method to thin-walled tube drawing with a fixed inner plug, modelling the plastic deformation zone using triangular rigid zones and accounting for frictional effects. Zhao et al. [15] investigated tube sinking using a novel mathematical approach. They determined the effective strain rate in a cylindrical coordinate system, expressing it as a two-dimensional vector. The power required for plastic deformation is calculated by term-by-term integration of the inner product of the strain rate vectors, and the total energy requirement is subsequently obtained as the sum of these terms. Rubio et al. [16] focused on the theoretical analysis of tube drawing processes with a fixed conical plug, applying and comparing the slab method and the UB Theorem. The study showed that analytical methods, particularly the UB Method, can provide results reasonably close to those obtained using FEM and experimental data while requiring substantially less computational effort. In Hungarian literature, a differential equation for fixed plug tube drawing is derived using the Slab Method (elementary equilibrium method). In this derivation, the drawing force is determined based on the axial and radial equilibrium formulated for an elemental annular slice of the tube wall as well as the Tresca flow criterion, which was described in detail by Tisza [17]. Building on this analytical approach, Szombathelyi and Krállics [18] applied the UB Theorem to define a kinematically permissible velocity field for the plastic zone of the tube wall, subsequently determining closed-form analytical formulas for internal deformation work, shear losses, and frictional losses through integration. Geleji [19] applied a modified slab method and an energetic approach, calculating with average values under the Tresca flow criterion. He treated the geometry of the plastic zone as a simplified truncated cone, thereby providing engineers with a rapidly computable relationship suitable for industrial design. Previously, Rubio [16] pointed out that with the spread of numerical and finite element (FE) methods, the use of analytical procedures was neglected in solving forming technology issues. However, as originally conceptualized by Siebel [20] through the foundational partitioning of total drawing energy into ideal deformation, external friction, and redundant shear components, an analytical method can be a good choice for modeling a process. This is because an analytical model can provide very fast solutions compared to finite element techniques and also ensure a better comprehension of the process.

**Figure 1:**
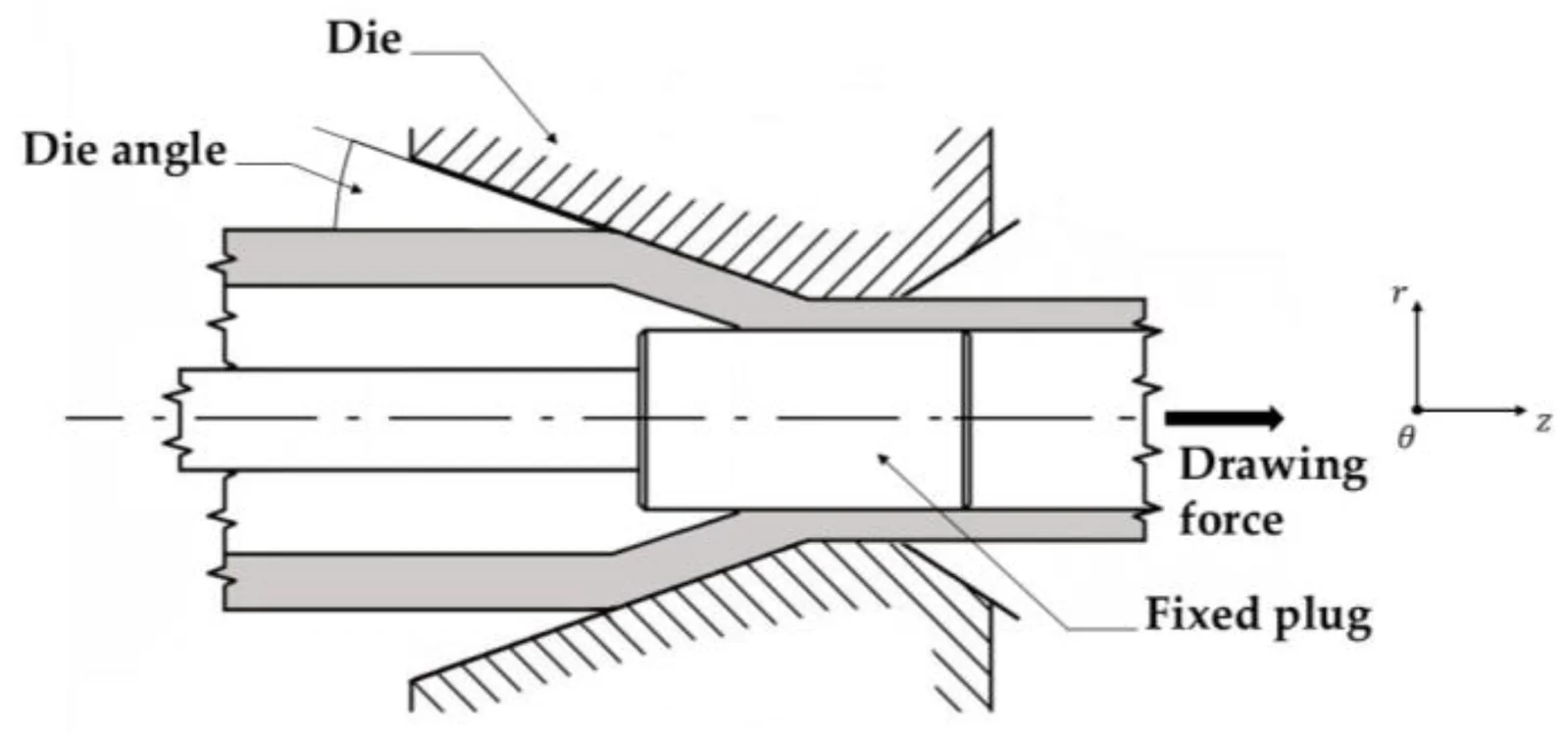
Schematic view of the tube drawing process using a fixed plug.

In the current work, a new analytical model of the deformation during fixed-plug tube drawing (FPTD) is presented (Figure 1). First, it is shown by FE simulations under von Mises flow conditions that the main deformation process within the die is simple shear along a shear plane. On that basis, we developed an analytical plasticity analysis which provided a new formula for the drawing tensile forces of the FPTD process. The obtained formula compares well with the results of the FE simulations used for reference, and also agrees well with our experimental results, both for the drawing force and for the crystallographic texture obtained at the end of the process.

## 2. Experiments

Fixed-plug tube drawing was carried out on steel tubes with an initial geometry of diameter D = 42 mm and tube wall thickness of T = 3.0 mm. The chemical composition of this structural steel tube is shown in Table 1.

**Figure 2:**
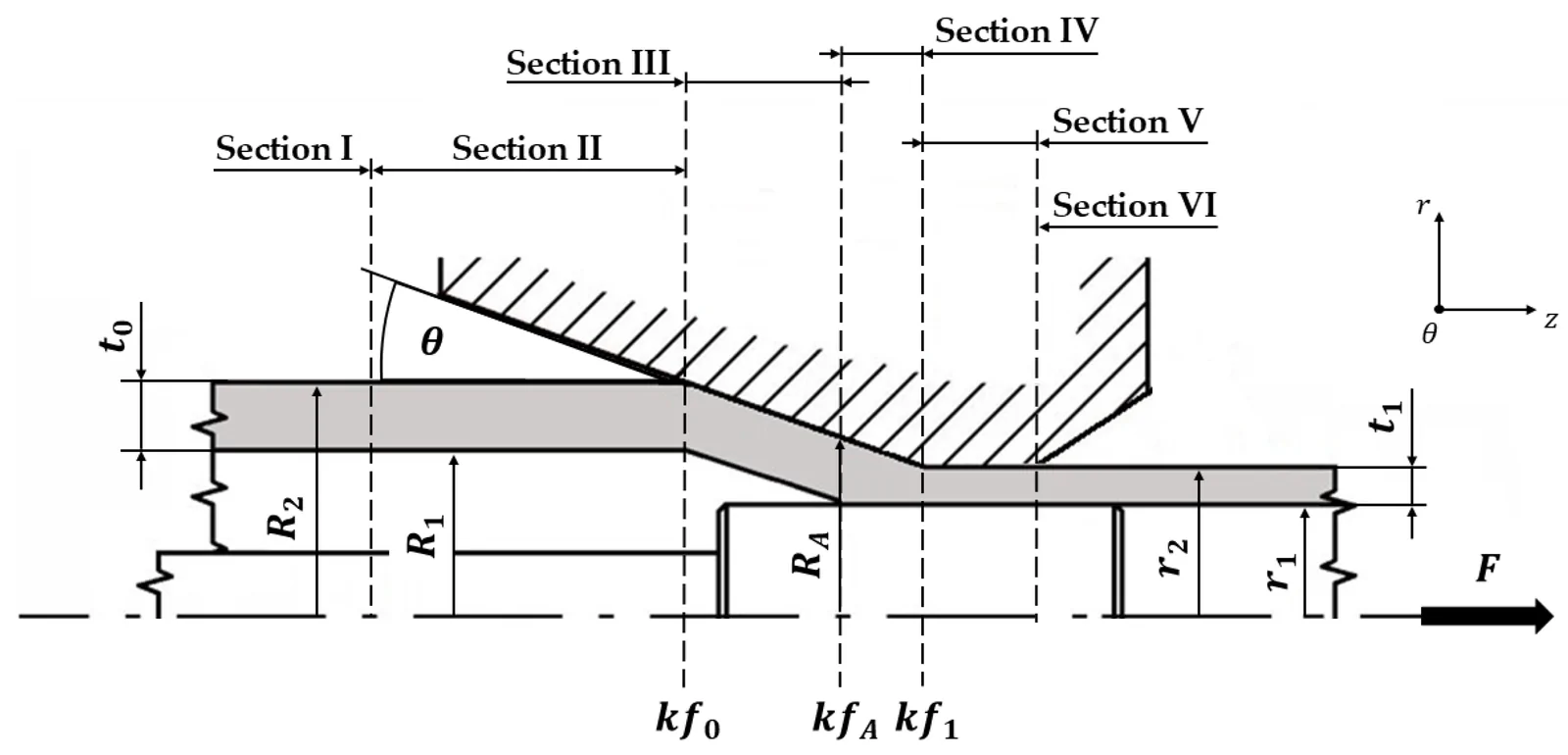
Geometric dimensions and tube drawing stages. In Figure 2 we name: Section I: start of the drawing without contact; Section II: start of bending without contact; Section III: stage considered as an empty draw, with the outer strand in contact; Section IV: shearing stage and plane with contact between the draw ring and the fixed plug; Section V: calibration phase with contact between the draw ring and the fixed plug; Section VI: exit phase without contact; *R*_2_ outer radius of the inlet tube (21 mm); *R*_1_ inner radius of the inlet tube (18 mm); *R_K_* medium outer radius (19.5 mm); *r*_2_ outer radius of the outlet tube (17.5 mm); *r*_1_ inner radius of the outlet tube (15 mm); *t*_0_ initial wall thickness (3 mm); *t*_1_ final wall thickness (2.5 mm); *R_A_* outer radius of the tube in the selection plane; *θ* semi-angle of the drawing die (13.3°); *k_f_*_0_ forming strength in the inlet cross-section (for Geleji formula); *k_fA_* forming strength in the selection plane (Geleji); *k_f_*_1_ forming strength in the outlet cross-section (Geleji).

The initial condition of tube was cold-formed and welded with annealing, followed by one thread drawing to produce a hard condition finished product. The outer diameter of the tube was reduced from 42 mm to 35 mm and the wall thickness from 3.0 mm to 2.5 mm (cross-sectional area reduction of 30.5%). The geometry of the tool had a semi-cone angle of *α* = 13.3°, similar to that in Yoshida’s [21] study. Figure 2 shows the geometric data for FTPD tube drawing, together with the locations of strength data used in the Geleji formula.

Tensile tests were performed on the initial tube material to determine its strain hardening behavior during plastic deformation on accredited material testing equipment in accordance with the ISO 6892-1 standard [22].

The crystallographic textures after the FPTD process were examined in the middle part of the cross-section of the tube by EBSD measurements on an AMETEK EDAX instrument equipped with an HIKARI camera. The sample was prepared first by polishing with wet grinding on SiC, an abrasive paper with grain sizes of 220, 320, 500, and 800. This was followed by a three-stage polishing sequence as follows:

- Using a Diapro Mol (3 μm) suspension on a 3 μm polishing cloth for 5 min at a pressure of 20 N,
- Using a Diapro Mol (1 μm) suspension on a 1 μm polishing pad for 5 min at a pressure of 15 N,
- Using an OPS Non-Dry (0.25 μm) suspension for 25 min at a pressure of 15 N.

The size of the EBSD map was 500 × 500 μm, obtained with a step size of 0.35 μm. The map contained 17.500 grains, permitting very good statistics for texture identification. The acquired EBSD data were analyzed with ATEX software v5.18 [23] to obtain complete pole figures.

## 3. Experimental Results

We carried out several tube drawing experiments with fixed-plug on different geometries of low-carbon steel tubes. A typical experiment was selected to analyze in detail in the present paper. This experiment was done at a pulling speed of 53 m/min with the geometrical parameters defined in the preceding section. After the initial transient stage, the drawing force stabilized at 11.25 tons.

**Figure 3:**
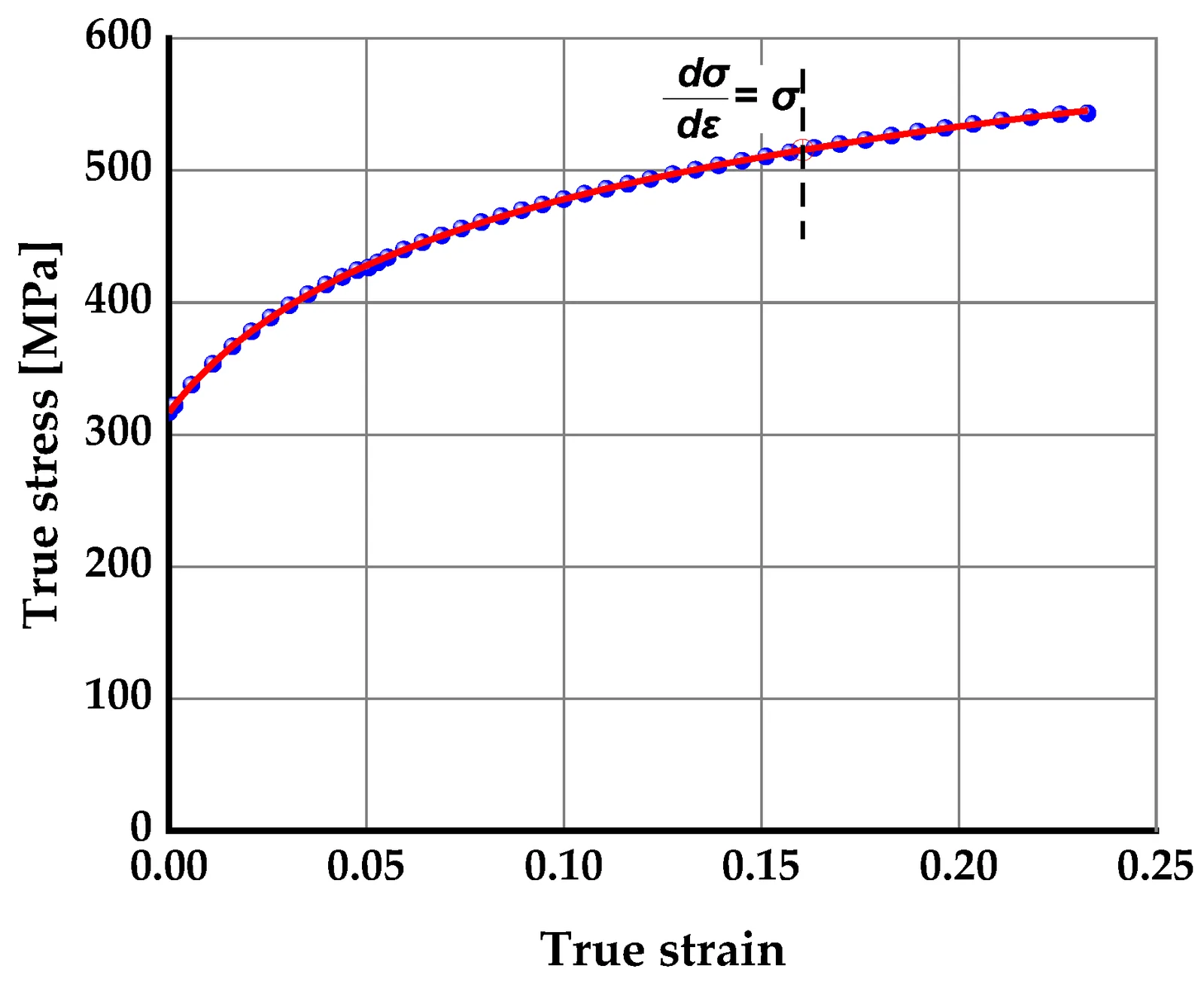
Experimental true stress–true strain curve (blue points) in tensile testing and the fitted function (red line). The vertical dotted line shows the point at which the Considère criterion is satisfied, which is the end-point of the uniform deformation of the sample.

An important element in the required drawing force is the strain hardening behavior of the material during drawing. This cannot be measured directly during drawing, so it was studied by separate uniaxial tensile testing. Three tensile samples were cut out from the initial tube parallel to the longitudinal axis of the tube, then the tensile tests were carried out at room temperature at 0.1 mm/s. The obtained force-displacement data were converted into true stress and true strain, which are needed in the simulations. There were small variations in the stress values of the three curves, so we used their average values. See Figure 3 for the measurement results.

For the FE simulation, it is better if an analytical function is used for the flow strength of the material as a function of strain. For this purpose, the measured curve was approximated by a recently developed new function [24], which is defined by four parameters in the following form:

(1) $$\sigma_{0}\left(\bar{\varepsilon }\right)=\sigma_{\mathrm{sat}}-\sigma_{\mathrm{sat}}{\left[\left(\frac{h\left(a-1\right)}{\sigma_{\mathrm{sat}}}\right)\bar{\varepsilon }+{\left(1-\frac{\sigma_{00}}{\sigma_{\mathrm{sat}}}\right)}^{1-a}\right]}^{\frac{1}{1-a}},\;\mathrm{where}\;a\;\neq \;1.$$

Here, *σ*_0_ is the equivalent flow stress as a function of the von Mises equivalent strain $\bar{\varepsilon }$, *σ_sat_* is its saturation value, and *σ*_00_ is the initial yield stress. The other parameters are *h* and *a*. The former is related to the rate of strain hardening, while the latter controls the shape of the curve. The values of the parameters were determined by a minimization process between the measured data and the data from the analytical function. Table 2 contains the parameter data obtained by the fitting process. As can be seen in Figure 3, a nearly perfect reproduction of the experimental curve was obtained; the average deviation between the analytical function and the measured points was only 0.2 MPa. Note that the experimental tensile test data are only valid before the Considère criterion is reached; from that point on, necking is developing in the sample. The reason to use the fitted function is that it allows the use of flow stress data in the FE simulations beyond that point.

**Figure 4:**
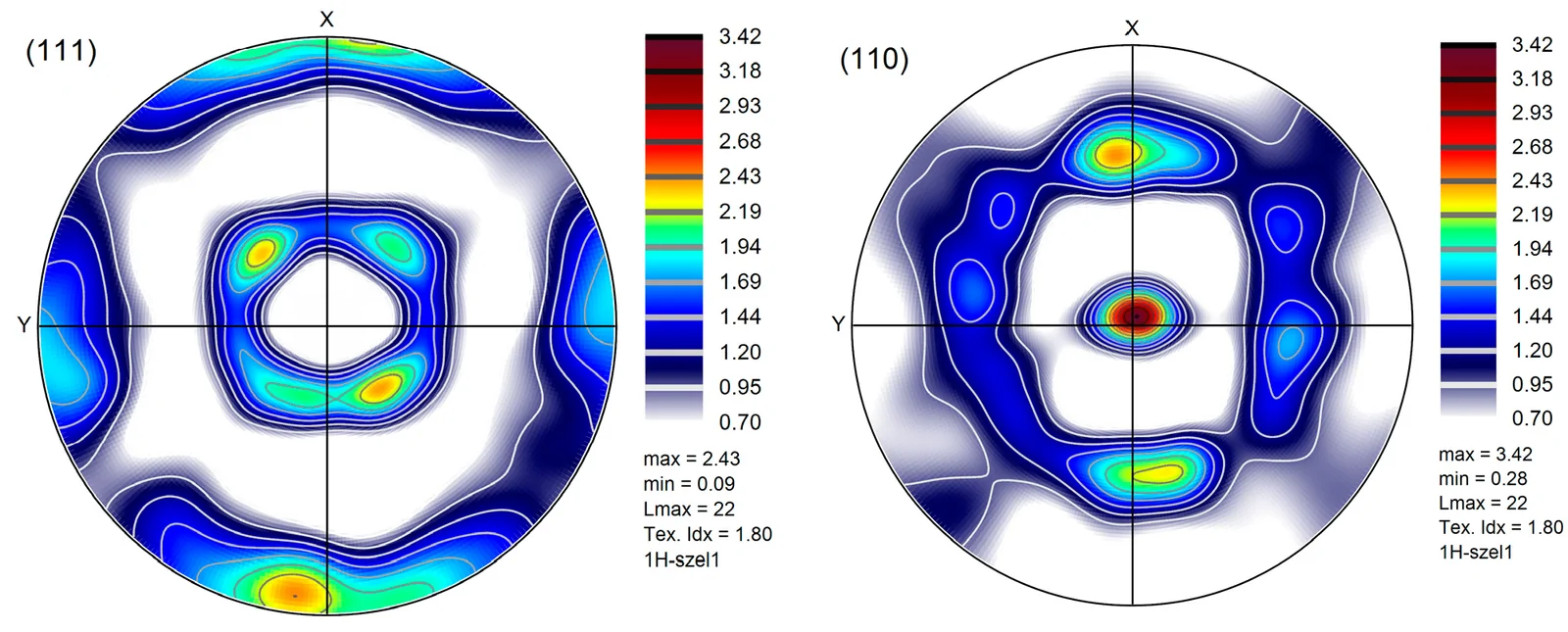
The crystallographic texture measured after drawing in the cross-section of the tube, in the middle part of the tube-wall, obtained from EBSD-analysis. The indicated *x* and *y* directions are the *r* and *θ* axes in the cylindrical reference system of the tube.

The microstructure was examined by EBSD measurements to obtain the crystallographic texture at the end of the tube drawing test in the cross-section of the drawn tube, in the middle part of the wall thickness. The measured texture is shown in Figure 4. It is presented in two pole figures in the {111} and {110} stereographic projections. The *x* and *y* axes indicated in the figure are the *r* and *θ* axes in the tube cylindrical reference system; the projection plane normal of the pole figures is the axis of the tube. As can be seen, the characteristic texture obtained is not fully symmetric—a slight rotation around the *y* axis by about 13° can be detected. The interpretation of the characteristics of this texture will be presented in Section 7 “Texture Analysis”, and further interpreted in the Section 8. It will help to confirm the analytical model that will be established in Section 6.

## 4. Finite Element Simulation Conditions

**Figure 5:**
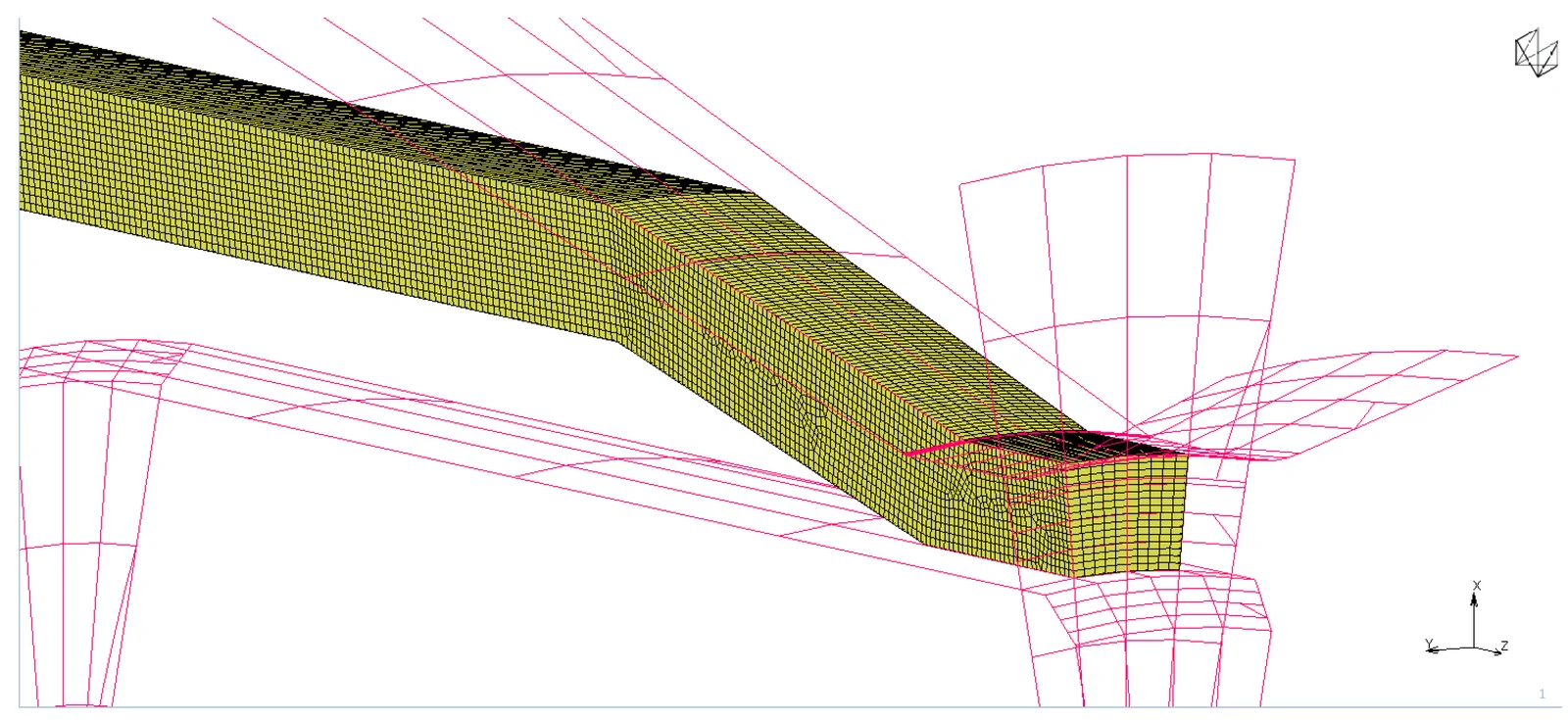
3D 10° segment of the finite element model (The elementary cells of the tube segment are shown in yellow, while the contours of the plug and the drawing die are shown in pink).

For the modelling of the cold drawing process, the general finite element MSC Marc software was used [25], in which contact problems of elastic-plastic materials deformed by small and large deformations can be solved. For tube drawing, we first created a 2D axisymmetric model, which was then transformed into a 3D model. In this way, we constructed a three-dimensional mesh (95,490 elementary cells) with an axially symmetric model, in which the drawing tools were considered rigid bodies and the drawn tube was defined as an elastic-plastic material. A 10° segment of the 360° full cross-section of the tube is shown in Figure 5.

The basic parameters of the drawing: the geometric dimensions of the tube, the drawing ring, and the fixed plug are given in Section 2 “Experiments”. The contact interaction between them was also defined in the model (touching or glued). 1000-time steps were used for the processing, with a time increment of 0.1 s. The friction between the tool and the tube as well as between the tube and the plug was considered as a Coulomb-type friction, using the same friction parameters everywhere. The value of the friction parameter was varied using five different coefficients: 0.05, 0.10, 0.15, 0.20, and 0.25. For strain hardening, Equation (1) was used for the flow stress as a function of strain with the parameters defined in Table 2. From the obtained results, the deformation velocity gradient field as well as the stress states were extracted, from which the tube-pulling tensile force could be calculated.

## 5. Results of Finite Element Simulations

**Figure 6:**
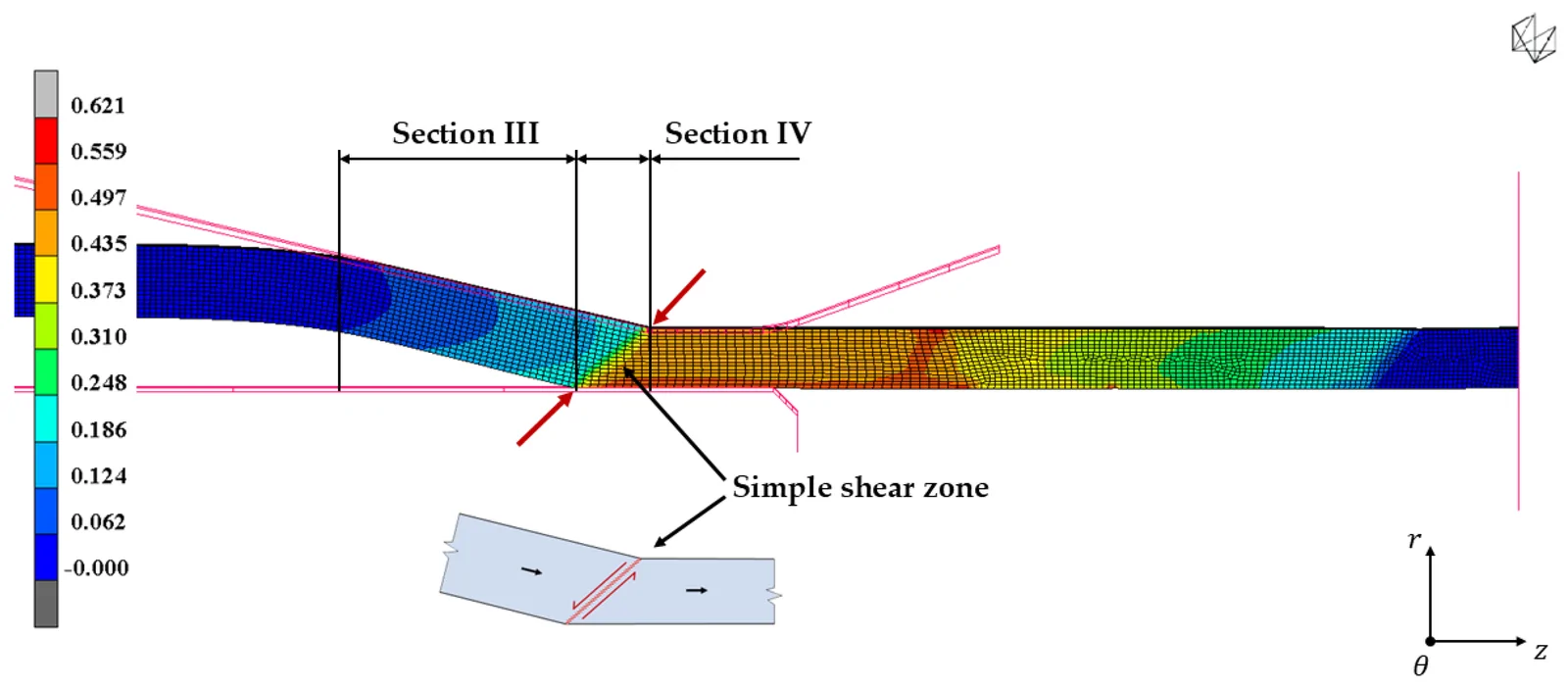
The accumulated strain distribution within the tube in the steady state of FPTD.

Figure 6 displays the map of the accumulated strain obtained in the steady state of the process. As can be seen, the total deformation was building up progressively. It was the most intense at the meeting part of Sections III and IV, where it appeared as a deformation zone inclined to the drawing direction at an angle α which is defined by the two corner regions of the specimen. It was found in this work that the main deformation mode in this zone locally is simple shear, the shear plane being the intersection plane between the two corner points of the deforming tube in this zone, as shown in the insert in Figure 6. When the material element crosses this zone, it deforms by simple shear and conserves the accumulated plastic deformation in it. This can be verified by the colors of the deformation level in Figure 6 after this zone. The shear zone is different from a shear band, because there is material flow across the shear zone, while there is no such flow in shear bands.

**Figure 7:**
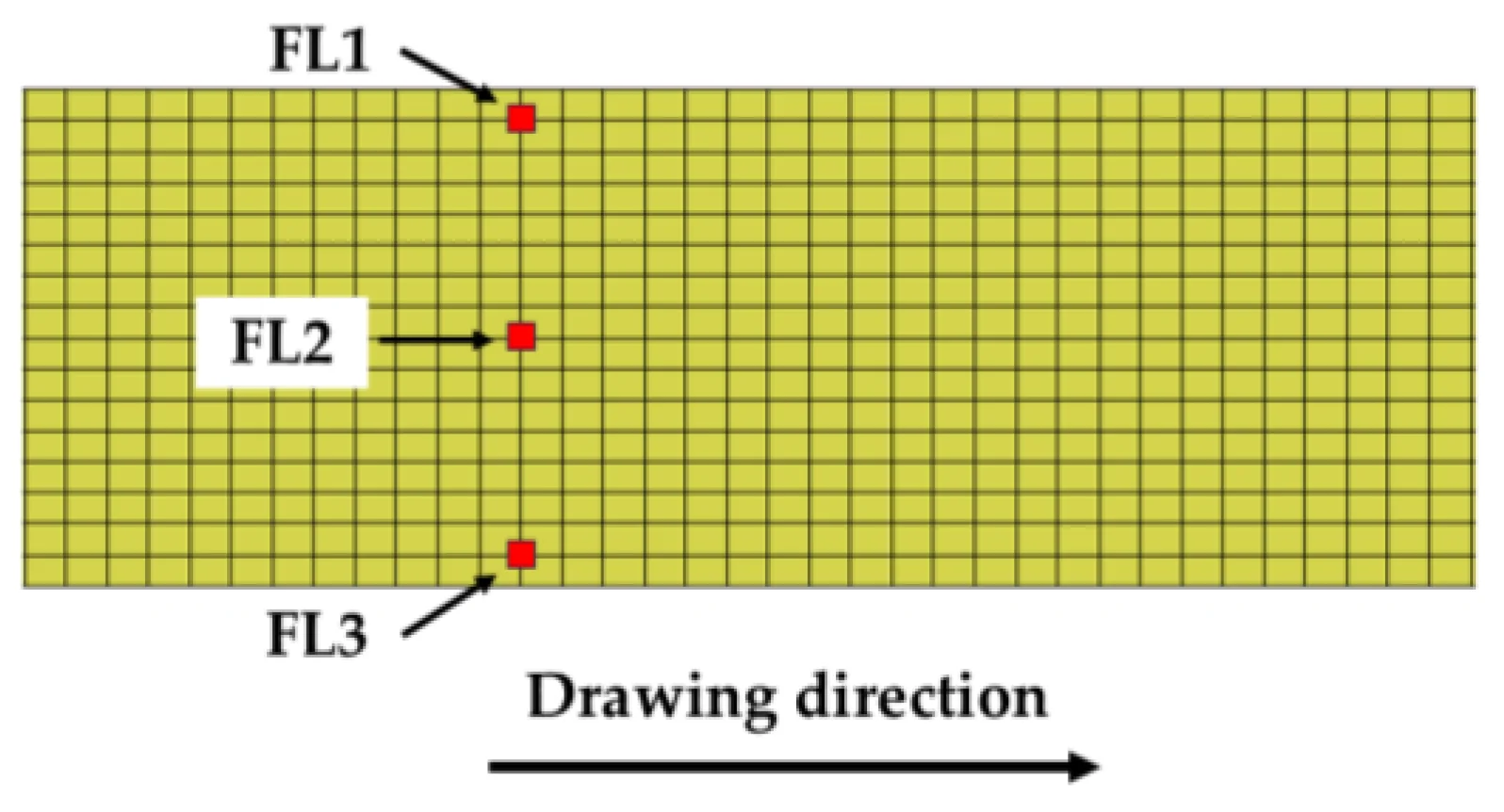
The positions of the starting points for the three selected flow lines before deformation.

One can also detect in Figure 6 a slight gradient in the amount of strain across the tube thickness, especially in Section III. For capturing this gradient, and for following the trajectory of the initial material points, three nodes were selected in the cross-section of the tube starting from the outer diameter, as shown in Figure 7. The flow lines that started at these points will be called FL1, FL2, and FL3 in the following pages.

### 5.1. Deformation Characteristics in FPTD

To represent the deformation field, the deformation gradient tensor components for each friction factor were extracted from the FE simulation along the three selected flow lines. As the simulation was done in cylindrical symmetry, the non-zero deformation gradient components are as follows:

(2) $$\underline{\underline{F}}=\left(\begin{array}{ccc} F_{\mathrm{rr}} & 0 & F_{\mathrm{rz}}\\ 0 & F_{\theta \theta } & 0\\ F_{\mathrm{zr}} & 0 & F_{\mathrm{zz}} \end{array}\right)$$

**Figure 8:**
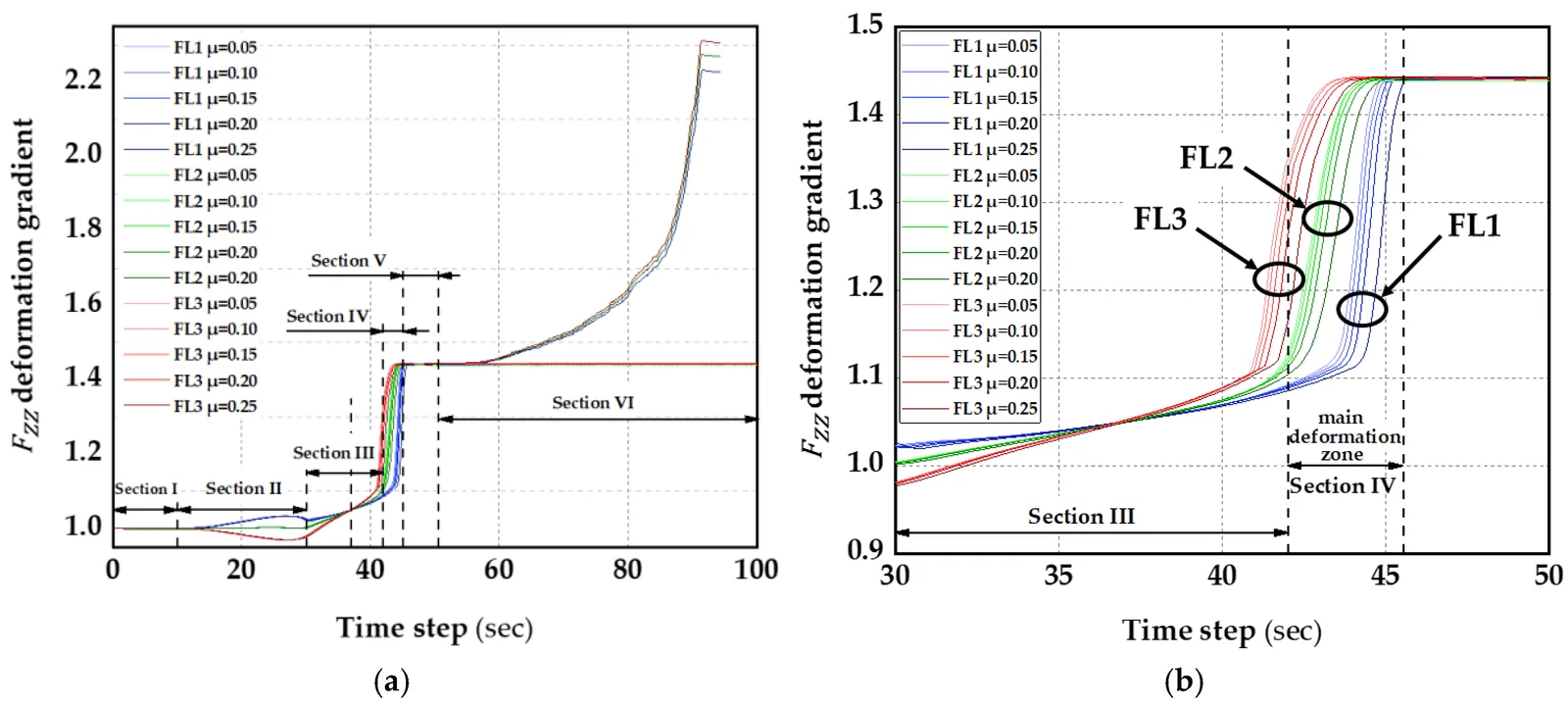
(**a**) Changes in the deformation gradient of the selected node *F_zz_* as a function of the friction coefficient for the three selected flow lines: FL1, FL2, FL3. (**b**) Same as (**a**), enlarged for the main deformation sections: III and IV. (The deformation gradient does not have dimension).

**Figure 9:**
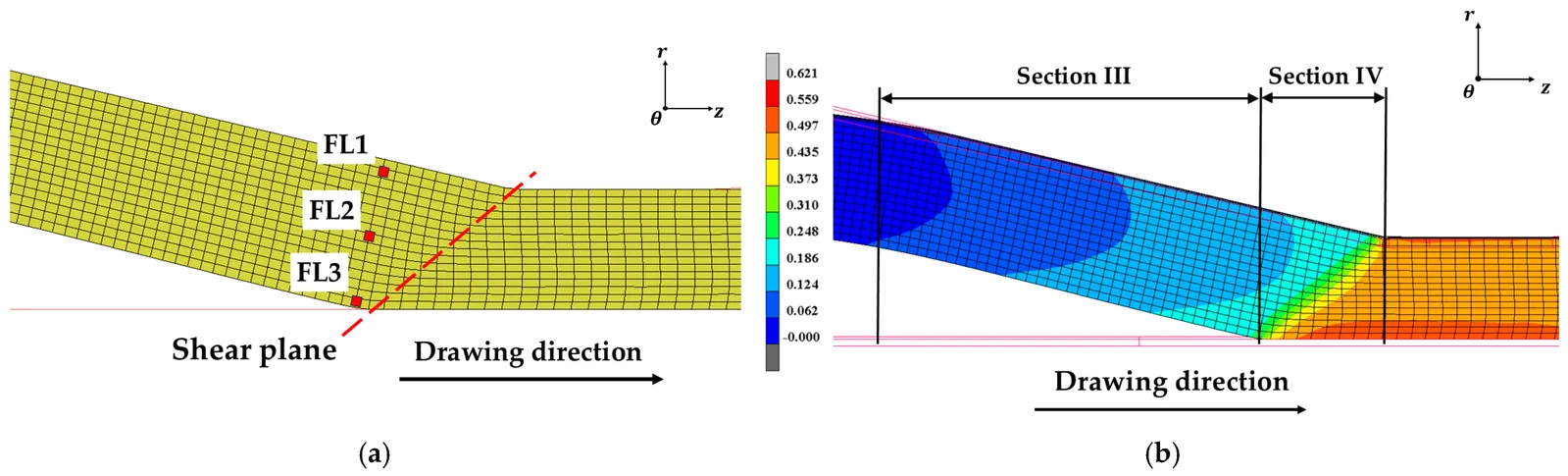
(**a**) Positions of the selected nodes (FL1, FL2, FL3) along their trajectory, just before flow line FL3 reaches zone IV. (**b**) Isovalue map of the von Mises strain in the steady-state.

The *F_zz_* deformation gradient component was selected for studying its variation along the three flow lines as a function of the values of the friction coefficient. The result for the whole passage is shown in Figure 8a. Figure 8b shows the results from the main deformation zones only. The evolution of the deformation gradient can be examined along the six stages of the drawing process (see the stages above in Figure 2). We recall that there is no plastic deformation in a component of the deformation gradient tensor if its value is 1.0, and for the velocity gradient if it is 0.0. In Figure 8b, it can be clearly seen that the main deformation zone is first reached on the flow line FL3, then on the middle flow line FL2, and finally on FL1. The main deformation zone seems to be a shear zone around the plane that connects the two corners of the tube in Section IV. See Figure 9a,b.

From the components of the deformation gradient tensor, one can determine the components of the velocity gradient tensor $\underline{\underline{L}}$ using its definition:

(3) $$\underline{\underline{L}}=\dot{\underline{\underline{F}}}\cdot {\underline{\underline{F}}}^{-1}$$

As the strain gradient tensor is known in successive points along the flow lines, the following numerical approximation was employed to compute $\underline{\underline{L}}$:

(4) $$\underline{\underline{L}}=\frac{\underline{\underline{F}}\left(t+∆t\right)-\underline{\underline{F}}\left(t\right)}{∆t}\cdot \;\frac{{\underline{\underline{F}}}^{-1}\left(t+∆t\right)+{\underline{\underline{F}}}^{-1}\left(t\right)}{2}$$

Similar to the deformation gradient tensor Equation (2), the velocity gradient tensor has the same form:

(5) $$\underline{\underline{L}}=\left(\begin{array}{ccc} L_{\mathrm{rr}} & 0 & L_{\mathrm{rz}}\\ 0 & L_{\theta \theta } & 0\\ L_{\mathrm{zr}}0 & 0 & L_{\mathrm{zz}} \end{array}\right)$$

**Figure 10:**
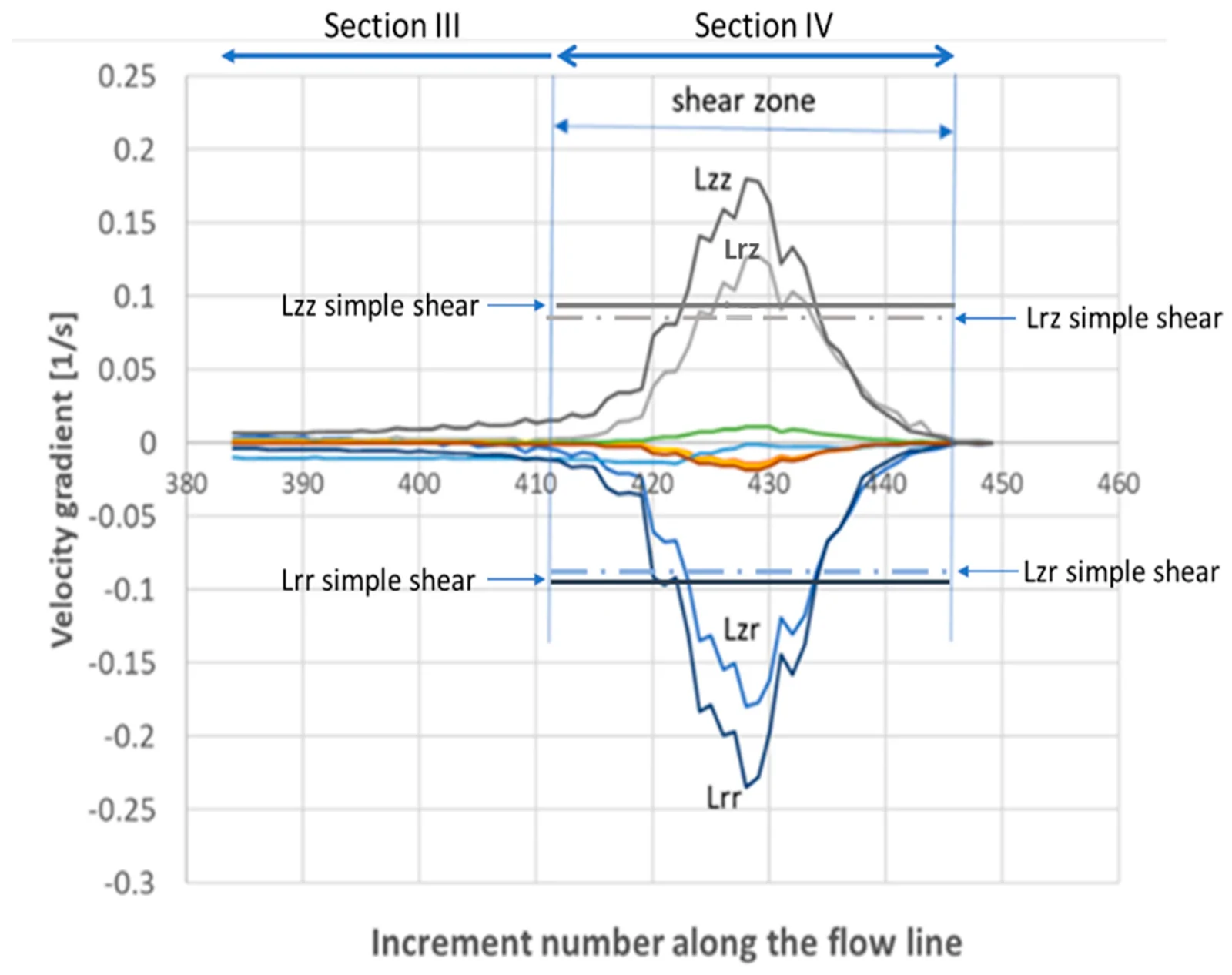
The FE-obtained velocity gradient components in the shear deformation zone (Section IV) along the flow line FL2 passing in the middle of the tube wall (varying curves). The constant values of the four non-zero velocity gradient components of the simple shear approach are presented by the horizontal lines (see the model in Section 6). The time difference between two increments is 0.1 s.

Figure 10 shows the velocity gradient components in Sections III and IV as a function of the movement of a material element along the flow line FL2 in the middle of the tube-wall. As can be seen in the shear deformation zone (section IV), there are four significantly large velocity gradient components in the FE simulation: the *L_zz_*, *L_rz_*, *L_zr_*, and *L_rr_*. The other components are relatively small. The four large components first progressively increase, then decrease to zero. Furthermore, the *L_zz_* and *L_rr_* components have exactly opposite values, satisfying the incompressibility condition of plastic deformation. This is not the case for the *L_rz_* and *L_zr_* components, where *L_zr_* is significantly larger than *L_rz_*. The consequence of this asymmetry will be discussed in Section 8 “Discussion”. We also plotted in the figure the values of the velocity gradient components of the analytical model (presented in Section 6), which are constant. They are indicated by the horizontal segments together with their values.

### 5.2. Drawing Forces

**Figure 11:**
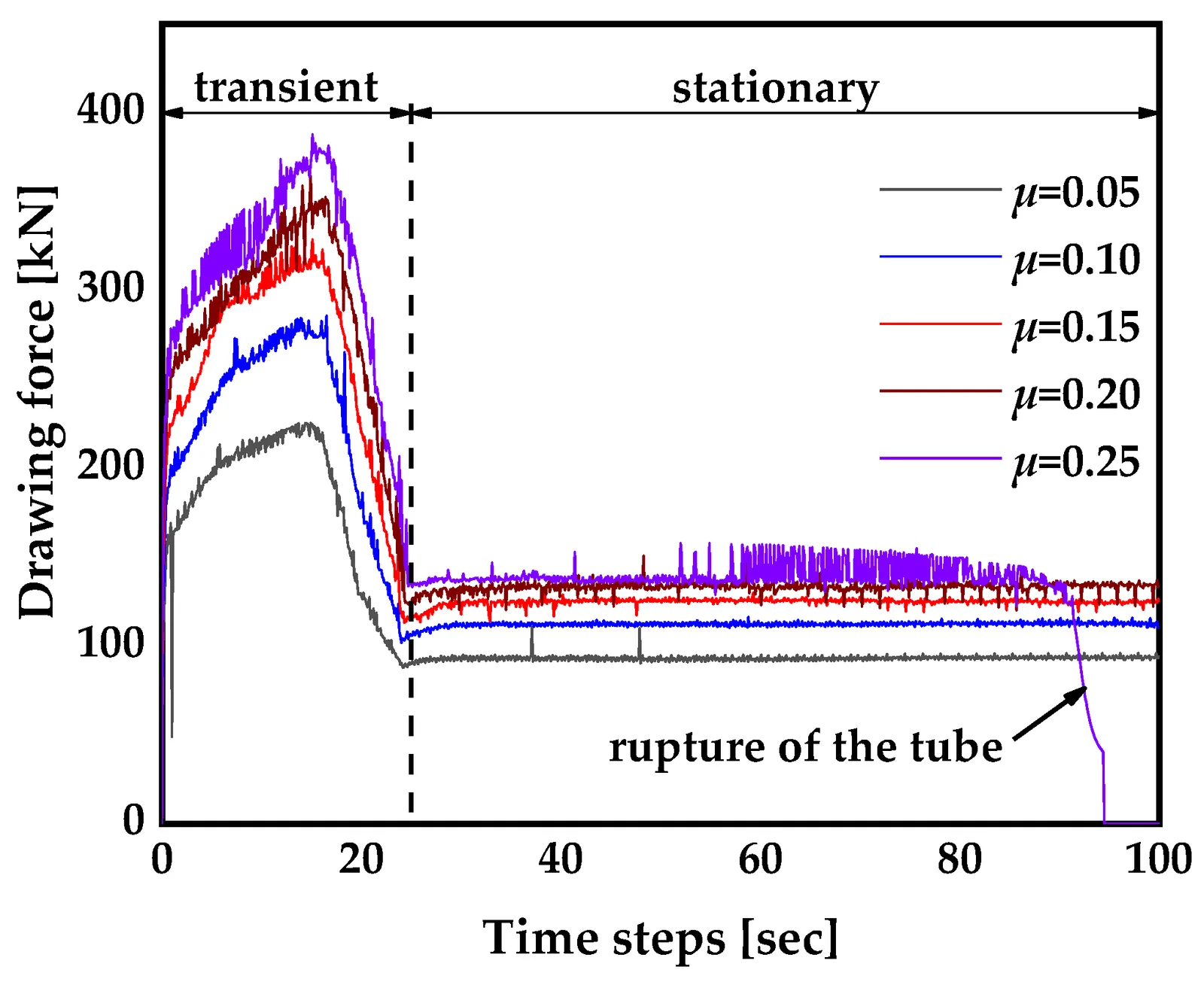
The FE-simulated changes in the drawing force as a function of the friction coefficient during the transient and stationary phases.

From the FE calculation, the variation of the drawing force to be exerted by the trolley on the tube along the *z*-axis as a function of the friction forces is illustrated in Figure 11, where it is clearly shown that the friction force increases the level of the drawing force.

In Figure 11, only the stationary phase of the evolution of the drawing force is relevant; the initial transient stage is artificial. It was necessary to artificially increase the strength of the tube at its leading part in order to avoid breaking the tube during the initial drawing stage. This is why the forces were so high at the beginning.

## 6. Analytical Modeling of the FPTD

**Figure 12:**
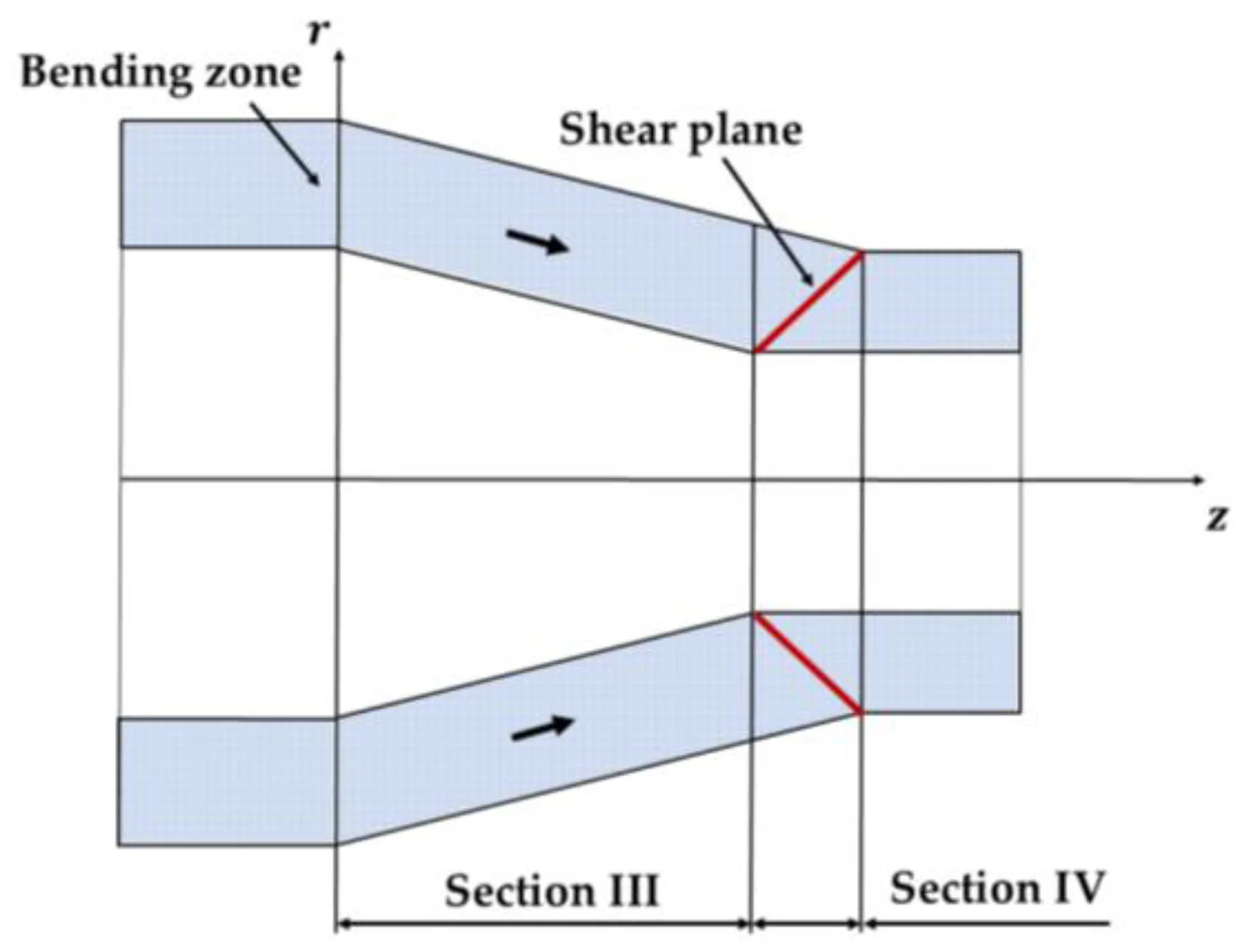
The principal deformation stages of the tube drawing process.

In this section, an analytical approach is established for the tube drawing process. It is based on the major information obtained from the FE simulation results. Our interest is on the main deformation zones, which is Sections III and IV. They are shown again Figure 12 (Section III is preceded by a bending zone in which the deformation is negligible). First, we examine the strain state in Sections III and IV. The stress state in Section IV will then be established with the help of the flow rule of plasticity.

### 6.1. Strain Calculation in Section III

**Figure 13:**
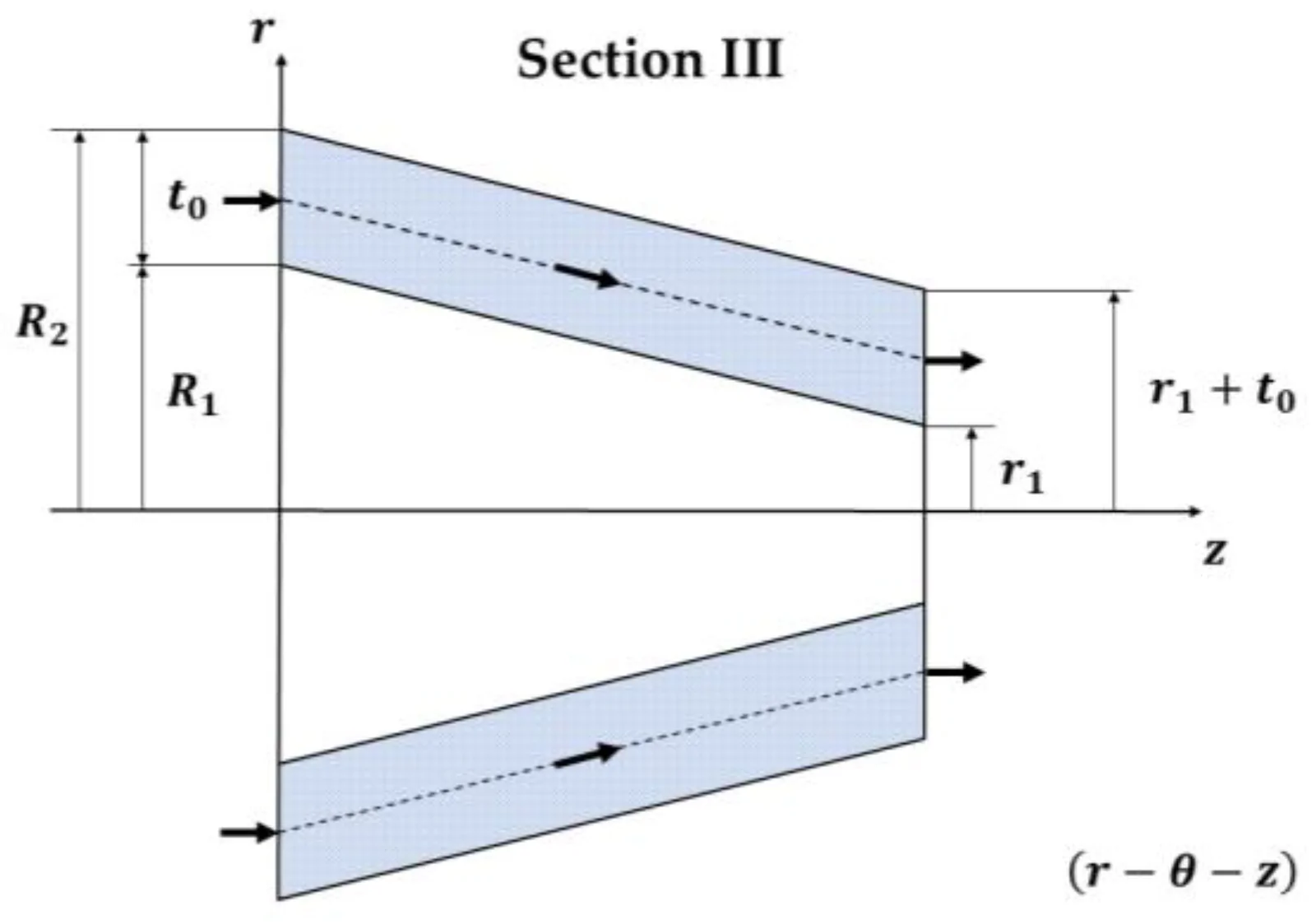
Geometry of the tube in Section III.

Figure 13 shows the geometry of the tube for the calculation in Section III of tube drawing. According to our FE simulations, there was no significant change in the wall thickness (*t*_0_) of the tube in Section III, so it is considered to be constant. There are no shear components in this stage, so the strain rate tensor is equal to the velocity gradient tensor. It has only two non-zero components: the hoop strain rate ${\dot{\varepsilon }}_{\theta \theta }$ and the axial (lengthening) strain rate ${\dot{\varepsilon }}_{\mathrm{zz}}$:

(6) $$\dot{\underline{\underline{\varepsilon }}}=\underline{\underline{L}}=\left(\begin{array}{ccc} 0 & 0 & 0\\ 0 & {\dot{\varepsilon }}_{\theta \theta } & 0\\ 0 & 0 & {\dot{\varepsilon }}_{\mathrm{zz}} \end{array}\right)$$

The hoop strain is defined by the change in the circumferential length, so one can write for a circular material element:

(7) $$d\varepsilon_{\theta \theta }=\frac{2\mathrm{r\pi}-2\left(r-\mathrm{dr}\right)\pi }{2\mathrm{r\pi}}=\frac{\mathrm{dr}}{r}$$

Therefore, the accumulated finite hoop strain at the exit of Section III, at a distance *r* from the center of the tube, is:

(8) $$\varepsilon_{\theta \theta }=\int_{R_{1}+r-r_{1}}^{r}\frac{\mathrm{dr}}{r}=\ln\frac{r}{R_{1}+r-r_{1}}=-\ln\left(1+\frac{R_{1}-r_{1}}{r}\right)$$

Here, for the value of the bottom limit of the integral, the constant tube thickness was used. The hoop strain is compression, because *R*_1_ > *r*_1_. Due to volume constancy in plastic deformation *ε_zz_* = −*ε_θθ_*. According to Equation (8), there is a gradient in the strain between the internal and external surfaces of the tube. It is generally very small. For example, for the present drawing parameters, one can readily calculate that it was only 2%. The maximum value is at the internal surface of the tube. The von Mises equivalent strain accumulated in Section III on a flow line ending at a position *r* is:

(9) $${\bar{\varepsilon }}_{\mathrm{vM}}=\frac{2}{\sqrt{3}}\ln\left(1+\frac{R_{1}-r_{1}}{r}\right)$$

### 6.2. Strain State in the Shear Zone

**Figure 14:**
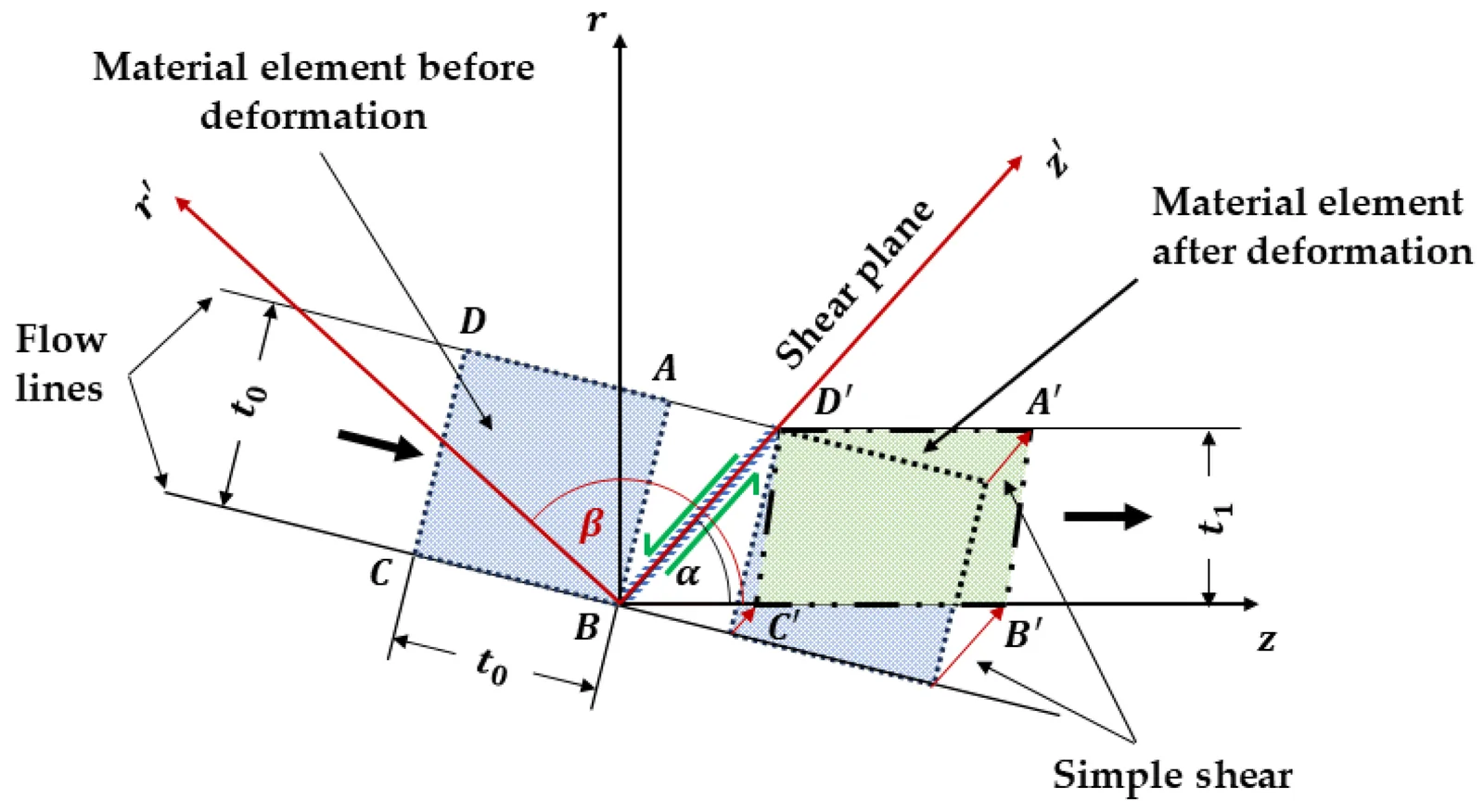
The shape of a material element before and after passing through the shear plane. The dashed area is the assumed plastic deformation zone.

We hypothesized that the simple shear occurs within a narrow zone situated along the intersection of the two flows, as shown in Figure 6. This type of deformation zone approximation was inspired from the flow line model described for NECAP (non-equal channel angular pressing) by Hasani and Toth [26]. Figure 14 shows how an initial material element denoted by the square *ABCD* is deforming by shear into *A’B’C’D’* as it passes through the shear plane. From the geometry in Figure 14, one can obtain a simple relation for the shear value as a function of the incoming and outgoing angles of the flow lines with respect to the shear plane:

*γ* = cot*α* + cot*β* (10)

This formula is actually the same as obtained by Lee, see Ref. [27].

**Figure 15:**
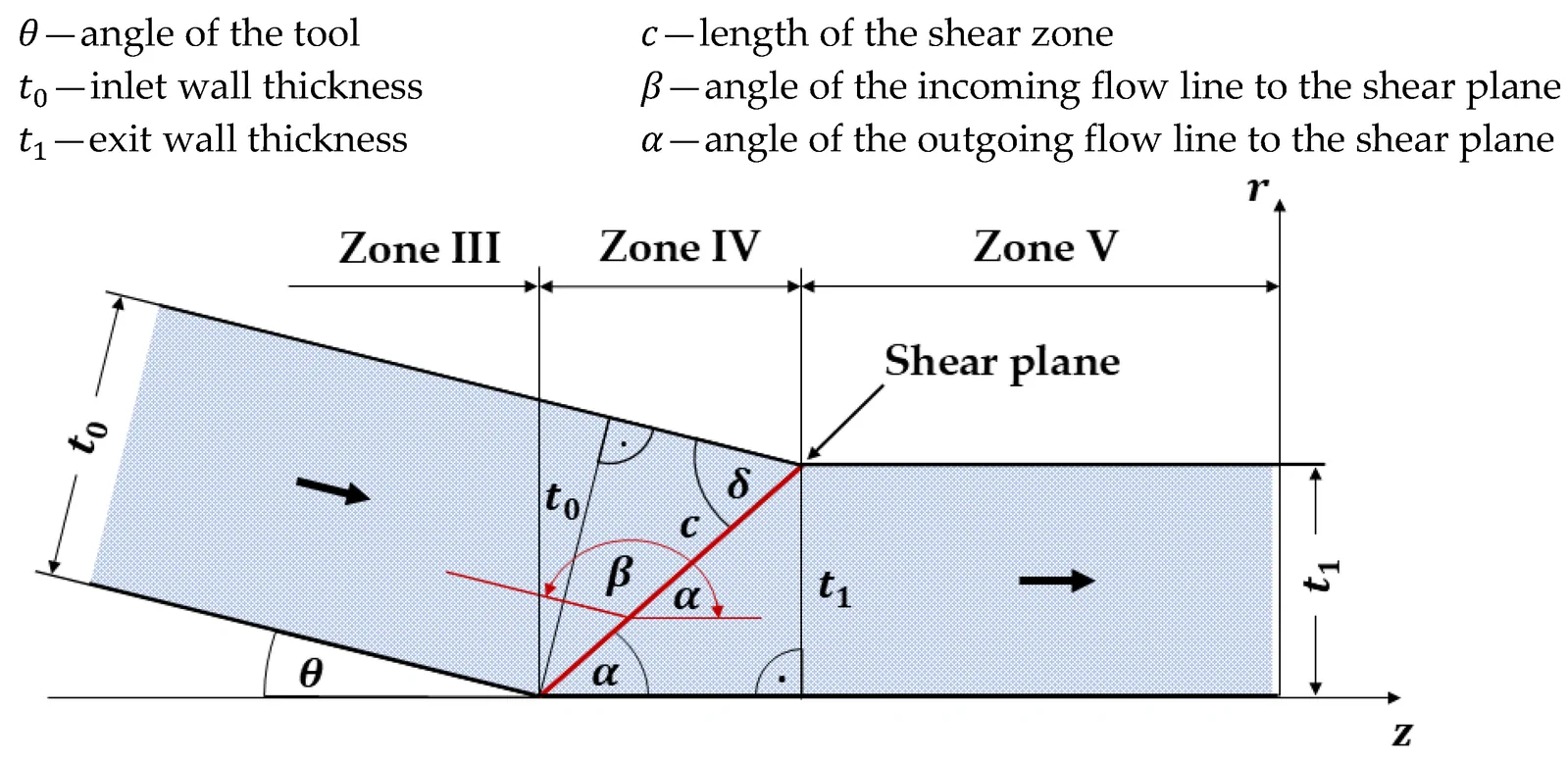
Schematic for the shear plane.

Figure 15 shows the notation used in the present work for the calculation of the shear strain.

Using the geometry in Figure 15, the *α* angle can be expressed as a function of *θ*, *t*_0_, and *t*_1_:

(11) $$\alpha =\arctan\left(\frac{\sin\theta }{\left(\frac{t_{0}}{t_{1}}\right)-\cos\theta }\right)$$

Now we need to obtain the velocity gradient of simple shear in the tube reference system defined by (*r*,*θ*,*z*) in Figure 14. It is the following simple matrix in the reference system (*r^′^*,*θ^′^*,*z^′^*), fixed to the shear plane:

(12) $${\underline{\underline{L}}}^{\prime }={\left(\begin{array}{ccc} 0 & 0 & 0\\ 0 & 0 & 0\\ \dot{\gamma } & 0 & 0 \end{array}\right)}_{r^{\prime },\theta^{\prime },z^{\prime }}$$

Here, $\dot{\gamma }$ is the shear deformation rate in the intersection plane. Its value is negative. We will denote it in the following as $-\dot{\left|\gamma \right|}$, to avoid possible sign issues. The transformation matrix between the two reference systems is:

(13) $$T=\left(\begin{array}{ccc} \cos\alpha & 0 & -\sin\alpha \\ 0 & 1 & 0\\ \sin\alpha & 0 & \cos\alpha \end{array}\right)$$

where the angle *α* is defined by Equation (11). Applying the transformation formula $\left(\underline{\underline{L}}=\underline{\underline{T}}\cdot {\underline{\underline{L}}}^{\prime }\cdot {\underline{\underline{T}}}^{T}\right)$, we obtain the following expression of the velocity gradient in the tube reference system:

(14) $$\underline{\underline{L}}=-\dot{\left|\gamma \right|}{\left(\begin{array}{ccc} -\sin\alpha \cos\alpha & 0 & -\sin^{2}\alpha \\ 0 & 0 & 0\\ \cos^{2}\alpha & 0 & \sin\alpha \cos\alpha \end{array}\right)}_{r,\theta ,z}$$

(Note that the angle *α* is negative here.) From this, we obtain the strain rate tensor:

(15) $$\dot{\underline{\underline{\varepsilon }}}=\frac{\underline{\underline{L}}+{\underline{\underline{L}}}^{T}}{2}=-\dot{\frac{\left|\gamma \right|}{2}}{\left(\begin{array}{ccc} -\sin2\alpha & 0 & \cos^{2}\alpha -\sin^{2}\alpha \\ 0 & 0 & 0\\ \cos^{2}\alpha -\sin^{2}\alpha & 0 & \sin2\alpha \end{array}\right)}_{r,\theta ,z}$$

It can be seen that there are four deformation components, the most important being the *rr* and the *zz*, where *rr* is compression and *zz* is extension. The values of the latter two components are opposite to each other due to volume constancy.

### 6.3. Stress State

The Cauchy stress tensor $\underline{\underline{\sigma }}$ during tube drawing is:

(16) $$\underline{\underline{\sigma }}={\left(\begin{array}{ccc} \sigma_{\mathrm{rr}} & 0 & \sigma_{\mathrm{rz}}\\ 0 & \sigma_{\theta \theta } & 0\\ \sigma_{\mathrm{zr}} & 0 & \sigma_{\mathrm{zz}} \end{array}\right)}_{r,\theta ,z}$$

First, the equilibrium equation will be solved without friction forces, which can be added after. Under the present cylindrical symmetry, it is the following:

(17) $$\frac{{\mathrm{d\sigma}}_{\mathrm{rr}}}{\mathrm{dr}}+\frac{\sigma_{\mathrm{rr}}-\sigma_{\theta \theta }}{r}=0$$

For the solution of this differential equation, we need another relation between the hoop stress and the radial stress. It can be obtained with the help of the associated flow rule and the von Mises flow criterion, which is adopted here. The flow rule is:

(18) $${\dot{\varepsilon }}_{\mathrm{ij}}=\dot{\lambda }\frac{\partial f\left(\underline{\underline{\sigma }}\right)}{{\partial \sigma }_{\mathrm{ij}}}$$

and the von Mises flow function:

(19) $$f\left(\underline{\underline{\sigma }}\right)=\frac{1}{6}\left[{\left(\sigma_{\mathrm{rr}}-\sigma_{\theta \theta }\right)}^{2}+{\left(\sigma_{\theta \theta }-\sigma_{\mathrm{zz}}\right)}^{2}+{\left(\sigma_{\mathrm{rr}}-\sigma_{\mathrm{zz}}\right)}^{2}+\frac{\sigma_{\mathrm{r\theta}}^{2}}{2}+\frac{\sigma_{\mathrm{\theta r}}^{2}}{2}+\frac{\sigma_{\mathrm{rz}}^{2}}{2}+\frac{\sigma_{\mathrm{zr}}^{2}}{2}+\frac{\sigma_{\mathrm{\theta z}}^{2}}{2}+\frac{\sigma_{\mathrm{z\theta}}^{2}}{2}\right]-\tau_{0}^{2}=0$$

Using the normality rule expressed in Equation (18) together with the von Mises flow criterion, the following equations connect the stress and strain rate states:

(20) $${\dot{\varepsilon }}_{\mathrm{rr}}=\dot{\lambda }\frac{\partial f}{{\partial \sigma }_{\mathrm{rr}}}=\frac{\dot{\lambda }}{3}\left(2\sigma_{\mathrm{rr}}-\sigma_{\theta \theta }-\sigma_{\mathrm{zz}}\right)=\dot{\frac{\left|\gamma \right|}{2}}\cdot \sin2\alpha$$

(21) $${\dot{\varepsilon }}_{\theta \theta }=\dot{\lambda }\frac{\partial f}{{\partial \sigma }_{\theta \theta }}=\frac{\dot{\lambda }}{3}\left(2\sigma_{\theta \theta }-\sigma_{\mathrm{rr}}-\sigma_{\mathrm{zz}}\right)=0$$

(22) $${\dot{\varepsilon }}_{\mathrm{zz}}=\dot{\lambda }\frac{\partial f}{{\partial \sigma }_{\mathrm{zz}}}=\frac{\dot{\lambda }}{3}\left(2\sigma_{\mathrm{zz}}-\sigma_{\mathrm{rr}}-\sigma_{\theta \theta }\right)=-\frac{\left|\dot{\gamma }\right|}{2}\sin2\alpha$$

(23) $${\dot{\varepsilon }}_{\mathrm{zr}}=\dot{\lambda }\frac{\partial f}{{\partial \sigma }_{\mathrm{zr}}}=\dot{\lambda }\sigma_{\mathrm{zr}}=-\dot{\frac{\left|\gamma \right|}{2}}\cdot \left(\cos^{2}\alpha -\sin^{2}\alpha \right)$$

From Equation (21), we obtain the hoop-stress:

(24) $$\;\sigma_{\theta \theta }=\frac{\sigma_{\mathrm{rr}}+\sigma_{\mathrm{zz}}}{2}$$

From Equations (20) and (24), the $\dot{\lambda }$ parameter is:

(25) $$\dot{\lambda }=\frac{\dot{\left|\gamma \right|}\sin2\alpha }{\left(\sigma_{\mathrm{rr}}-\sigma_{\mathrm{zz}}\right)}$$

Using this in Equation (23), the *σ_zr_* stress components is:

(26) $$\sigma_{\mathrm{zr}}=\frac{1}{4}\left(\sigma_{\mathrm{rr}}-\sigma_{\mathrm{zz}}\right)\cdot \left(\tan\alpha -\cot\alpha \right)$$

Substituting Equations (24) and (26) into equation (19), we get:

|*σ_rr_* − *σ_zz_*| = *Hτ*_0_ (27)

Here, the notation *H* is introduced:

(28) $$H=\frac{2}{\sqrt{1+\frac{1}{4}\cdot {\left(\tan\alpha -\cot\alpha \right)}^{2}}}$$

Since we are pulling in the direction of the *z*-axis, *σ_zz_* is positive while *σ_rr_* is negative. Therefore:

*σ_zz_* = *Hτ*_0_ − |*σ_rr_*| (29)

Returning to the equilibrium equation (Equation (17)), and using Equations (24) and (29), we get:

(30) $$\frac{{\mathrm{d\sigma}}_{\mathrm{rr}}}{\mathrm{dr}}+\frac{-\frac{H\tau_{0}}{2}}{r}=0$$

This leads to the following integral:

(31) $$\int {\mathrm{d\sigma}}_{\mathrm{rr}}=\int \frac{H\tau_{0}}{2r}\mathrm{dr}$$

**Figure 16:**
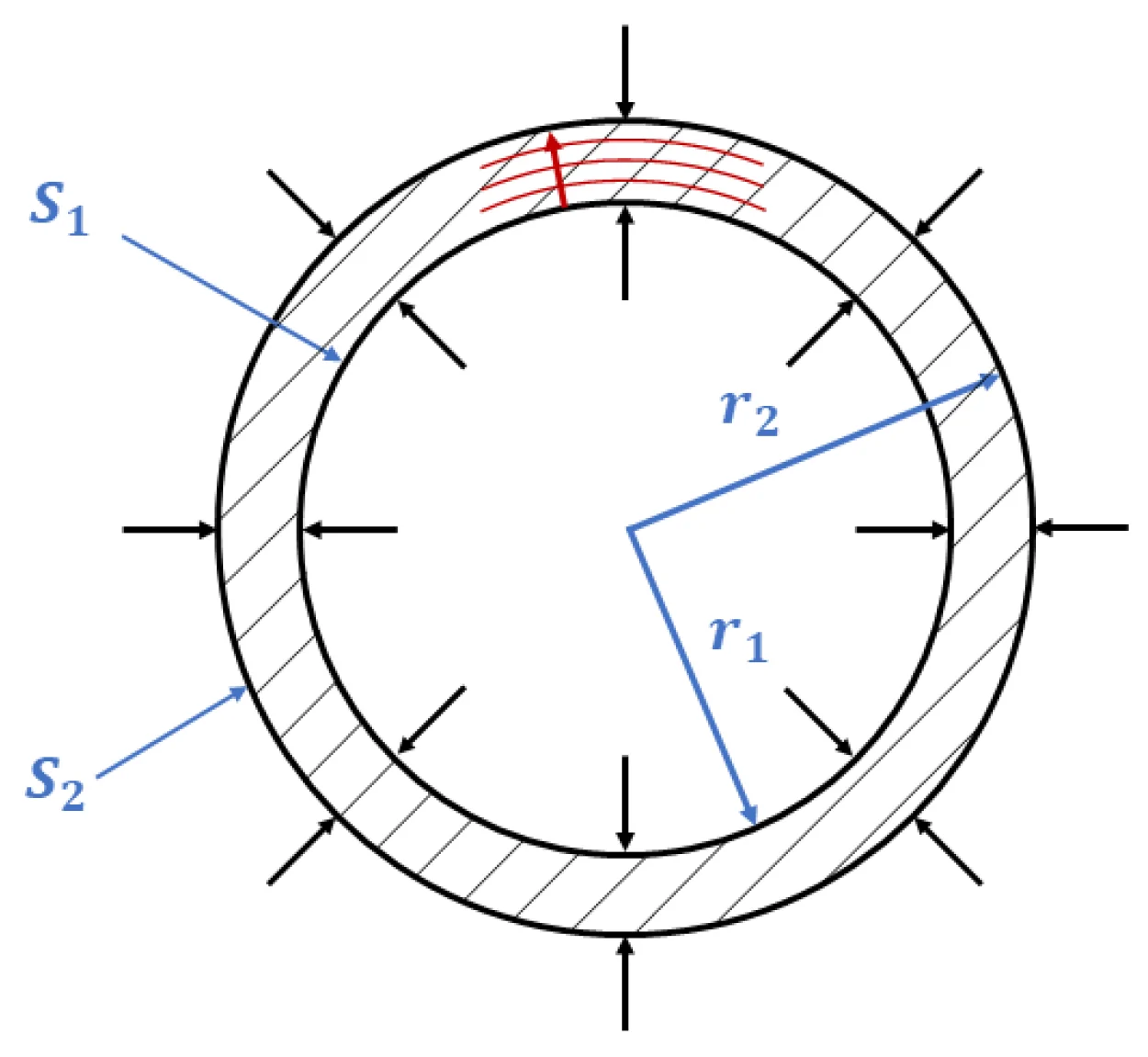
Schematic illustration for the radial stress.

The solution of the integral is:

(32) $$\sigma_{\mathrm{rr}}+C=\frac{H\tau_{0}}{2}\ln r$$

where *C* is an integration constant. In order to determine the value of the integration constant, we consider the boundary conditions for the *σ_rr_* component, meaning the internal surface of the tube is compressed by the mandrel while its external surface is pressed by the die. These two forces are in equilibrium, so we can write for a small segment (∆*θ*) of the tube:

(33) $$\int_{{∆S}_{1}}\sigma_{\mathrm{rr}}\mathrm{dS}=\int_{{∆S}_{2}}\sigma_{\mathrm{rr}}\mathrm{dS}$$

where ∆*S*_1_ is the internal surface area and ∆*S*_2_ is the corresponding external surface area (Figure 16):

For small ∆*θ*, Equation (33) can be written as:

(34) $$\sigma_{\mathrm{rr}}\left(r_{1}\right)\cdot r_{1}\cdot ∆\theta \cdot l=\sigma_{\mathrm{rr}}\left(r_{2}\right)\cdot r_{2}\cdot ∆\theta \cdot l\to \frac{\sigma_{\mathrm{rr}}\left(r_{1}\right)}{\sigma_{\mathrm{rr}}\left(r_{2}\right)}=\frac{r_{2}}{r_{1}}$$

Using this relation in Equation (32), the integration constant *C* can be derived:

(35) $$C=\frac{H}{2}\tau_{0}\frac{r_{2}\ln r_{1}-r_{1}\ln r_{2}}{r_{2}-r_{1}}$$

We then obtain the full expression of the radial stress component from Equation (32):

(36) $$\sigma_{\mathrm{rr}}=\frac{H}{2}\tau_{0}\cdot \left(\ln r-\frac{r_{2}\ln r_{1}-r_{1}\ln r_{2}}{r_{2}-r_{1}}\right)$$

Finally, with Equation (29), *σ_zz_* can be expressed only as a function of the radius:

(37) $$\sigma_{\mathrm{zz}}=\frac{H\tau_{0}}{2}\left[2-\ln r+\frac{r_{2}\ln r_{1}-r_{1}\ln r_{2}}{r_{2}-r_{1}}\right]$$

We introduce the following notation:

(38) $$G=2+\frac{r_{2}\ln r_{1}-r_{1}\ln r_{2}}{r_{2}-r_{1}}$$

Then, Equation (37) becomes:

(39) $$\sigma_{\mathrm{zz}}=\frac{H}{2}\tau_{0}\cdot \left(G-\ln r\right)$$

The drawing force can be determined by integrating *σ_zz_* over the cross section of the tube wall:

(40) $$F_{z}=H\int_{r_{1}}^{r_{2}}\sigma_{\mathrm{zz}}\cdot 2\mathrm{r\pi dr}=\frac{H}{2}\tau_{0}\int_{r_{1}}^{r_{2}}\cdot \left(G-\ln r\right)\cdot 2\mathrm{r\pi dr}$$

$\int_{r_{1}}^{r_{2}}r\ln r\mathrm{dr}$ can be determined by partial integration:

(41) $$\int_{r_{1}}^{r_{2}}r\ln r\mathrm{dr}=\frac{r_{2}^{2}}{2}\ln r_{2}-\frac{r_{2}^{2}}{4}-\frac{r_{1}^{2}}{2}\ln r_{1}+\frac{r_{1}^{2}}{4}$$

So, we obtain the frictionless drawing force requirement:

(42) $$F_{z}=\frac{H\tau_{0}\pi }{2}\left[r_{1}^{2}\ln r_{1}-r_{2}^{2}\ln r_{2}+\left(r_{2}^{2}-r_{1}^{2}\right)\left(\frac{5}{2}+\frac{r_{2}\ln r_{1}-r_{1}\ln r_{2}}{r_{2}-r_{1}}\right)\right]$$

To account for the effect of friction, Coulomb’s law was applied in this work. Friction was considered between the die and the tube as well as between the tube-plug contact surfaces, which were acting only above and below the plastic deformation zone. The length of the plastic zone in the axial direction is:

(43) $$l_{p}=\frac{t_{1}}{\tan\alpha }$$

Considering the friction on both surfaces, with different friction coefficients (*μ*_1_, *μ*_2_), the friction force is:

(44) $$F_{s}={\mu_{2}\cdot \sigma }_{\mathrm{rr}}\left(r_{2}\right)\cdot A_{2}+{\mu_{1}\cdot \sigma }_{\mathrm{rr}}\left(r_{1}\right)\cdot A_{1}$$

The surfaces of the friction zones are:

(45) $$A_{1}=2r_{1}\pi \frac{t_{1}}{\tan\alpha }\;,\;A_{2}=2r_{2}\pi \frac{t_{1}}{\tan\alpha }$$

Using Equations (36), (44) and (45), the friction force for different friction coefficients is:

(46) $$F_{s}=\tau_{0}\frac{H{\mathrm{\pi t}}_{1}}{\tan\alpha }\left\{\mu_{2}r_{2}\left(\ln r_{2}-\frac{r_{2}\ln r_{1}-r_{1}\ln r_{2}}{r_{2}-r_{1}}\right)+\mu_{1}r_{1}\left(\ln r_{1}-\frac{r_{2}\ln r_{1}-r_{1}\ln r_{2}}{r_{2}-r_{1}}\right)\right\}$$

When the friction coefficients can be considered equal between the inner and outer contacts of the tube, the friction force is:

(47) $$F_{s}=\mu \tau_{0}\frac{\mathrm{H\pi}t_{1}}{\tan\alpha }\cdot \left[r_{2}\cdot \ln r_{2}+r_{1}\cdot \ln r_{1}-\left(\frac{r_{2}+r_{1}}{r_{2}-r_{1}}\right)\cdot \left(r_{2}\ln r_{1}-r_{1}\ln r_{2}\right)\right]$$

Finally, the total drawing force requirement for equal friction coefficients is:

(48) $$F\;=\;F_{z}\;+\;F_{s}\;=\;\frac{H\tau_{0}\pi }{2}\left[r_{1}^{2}\ln r_{1}-r_{2}^{2}\ln r_{2}+\left(r_{2}^{2}-r_{1}^{2}\right)\left(\frac{5}{2}+\frac{r_{2}\ln r_{1}-r_{1}\ln r_{2}}{r_{2}-r_{1}}\right)\right]+\;\mu \tau_{0}\frac{\mathrm{H\pi}t_{1}}{\tan\alpha }\cdot \left[r_{2}\cdot \ln r_{2}+r_{1}\cdot \ln r_{1}-\left(\frac{r_{2}+r_{1}}{r_{2}-r_{1}}\right)\cdot \left(r_{2}\ln r_{1}-r_{1}\ln r_{2}\right)\right]\;\mathrm{where}\;H=\frac{2}{\sqrt{1+\frac{1}{4}\cdot {\left(\tan\alpha -\cot\alpha \right)}^{2}}}\;\mathrm{with}\;\alpha =\arctan\left(\frac{\sin\theta }{\left(\frac{t_{0}}{t_{1}}\right)-\cos\theta }\right)$$

## 7. Texture Analysis

In the following, the crystallographic texture will be analyzed both experimentally and by simulation, which is very sensitive to the strain path. Moreover, it is a 3D quantity, so particularly suitable to verify the validity of our proposed tube-drawing deformation model.

For the simulation of the texture evolution, the Viscoplastic Self-Consistent (VPSC) polycrystal plasticity model was used in its numerical VPSC5 version [28]. This code was updated for using the Kalidindi-type of self and latent hardening approach [29], in order to use the simulated tensile stress-strain curve presented in Section 3 “Experimental results”.

The VPSC code requires as input:

- A polycrystal comprised of individual grains, representing the initial texture: 5000 random grain orientations were generated for this purpose.
- The list of possible slip systems: the {110}<111>, {112}<111>, and {123}<111> slip system families were considered, which are well-known for deformation of b.c.c. metals.
- The velocity gradient tensor for each strain increment. The strain increment was 0.01 von Mises equivalent strain in the new simple shear model and had varying values not exceeding 0.01 in the FE simulation.
- A choice for the viscoplasticity parameter of dislocation slip: The value of m = 0.05 was employed.
- A choice for the value of the interaction parameter in the stress-strain localization equation (see more about it in Ref. [30]): The value of 1/*m* = 20 was used, which is the Tangent VPSC approach (*m* is the viscosity exponent for dislocation glide).

Two simulations were carried out:

(i) One used the velocity gradient obtained directly from the FE simulation (see in Section 5 “Results of finite element simulations”), starting at a middle-point of the tube wall before entering the die and following the flow line in the deformation zone.
(ii) The other used the analytical velocity gradient tensors developed in this work for the two main deformation stages during the tube-drawing: Equation (6) for Section III and Equation (14) for Section IV.

**Figure 17:**
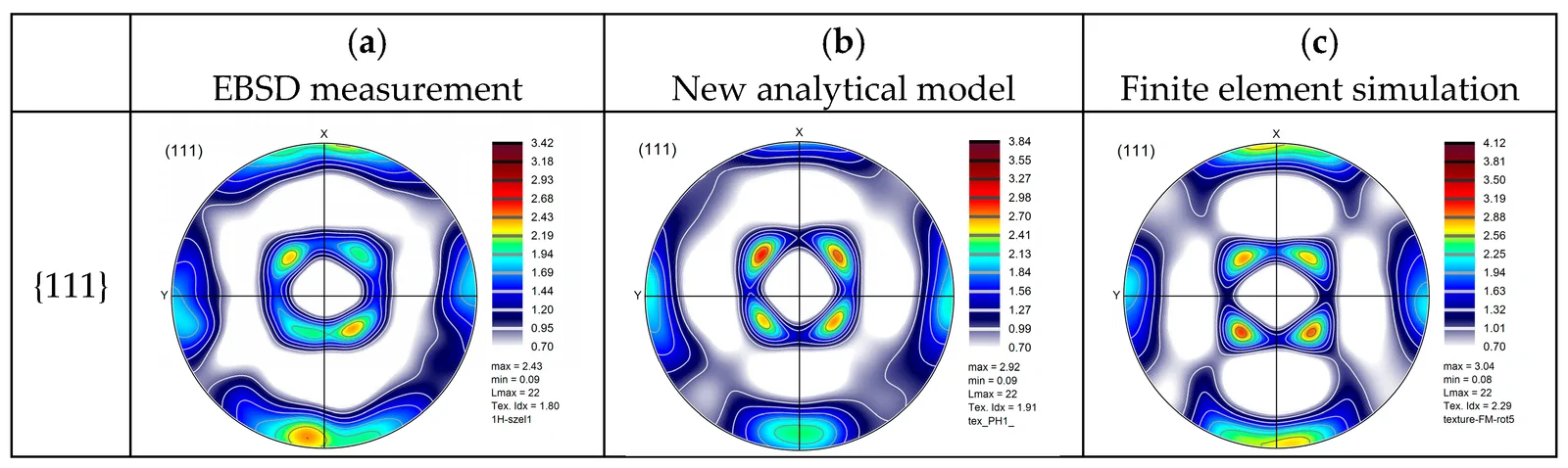

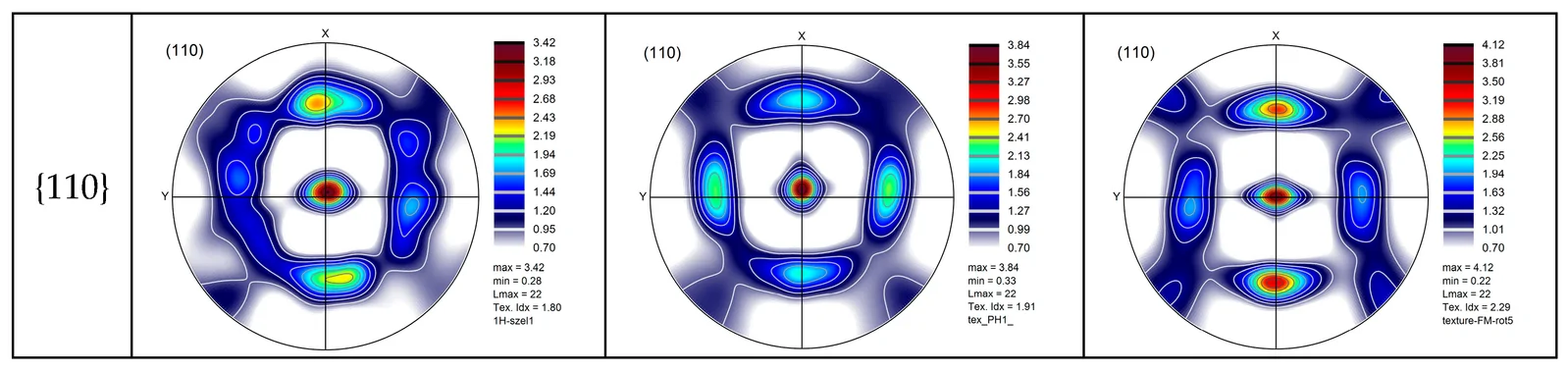
Simulated and experimental textures presented in {111} and {110} pole figures. (Axis *x* is the *r* radial axis of the tube, and *y* is the tangential *θ* axis.).

Both the experimental and simulated textures are presented in Figure 17 in the form of {111} and {110} pole figures projected in the cross-section of the tube. The reference system is defined by its vertical axis parallel to the radial axis *r* of the tube. The horizontal axis is the *θ* tangential axis. For comparison, the experimental texture is displayed again in Figure 17a, for the analytical model in Figure 17b, and for the FE simulation in Figure 17c. As can be seen, there is a good visual agreement between all three textures. The quality of the agreement between experiment and simulation can be quantified by the scalar texture correlation index, which is defined by:

(49) $$Q=\frac{\int f_{1}\left(g\right){\cdot f}_{2}(g)\mathrm{dg}}{\sqrt{\int {f_{1}\left(g\right)}^{2}\mathrm{dg}}\cdot \sqrt{\int {f_{2}\left(g\right)}^{2}\mathrm{dg}}},\;\mathrm{with}\;\mathrm{dg}=d\varphi_{1}\cdot \mathrm{d\varphi}\cdot d\varphi_{2}\cdot \sin\left(\varphi \right)$$

Here *f*_1_(*g*) and *f*_2_(*g*) are the two orientation distribution functions (ODFs) of the textures to compare. *φ*_1_, *φ*, *φ*_2_ are the Euler angles, *dg* is the unit volume in Euler space. Integration is done in the full Euler space. Perfect agreement between two textures leads to *Q* = 1.0, and total disagreement is *Q* = 0.0. Here, *Q* will be multiplied by 100 to express the agreement in percentage unit. The ATEX software v5.18 [23] was used to compute *Q*. The obtained agreements between simulations and experiment are good: 92% for the analytical approach, and 91% for the FE simulation. These values are actually very high if one considers that the agreement is obtained in a 3D space!

Looking more closely at the three textures presented in Figure 17, the following observations are important.

(i) The analytical model reproduces the small rotation of the texture around the *y* (or *θ*) axis (about 13°), while in the FE simulation, the pole figures are symmetric.
(ii) While both textures are reproducing well the experimental texture, the shapes of the pole figures are closer to the experimental ones in the analytical model.
(iii) At the same time, the maximum intensity values are better reproduced by the FE simulation.

An interpretation of the small texture rotation, and its absence in the FE simulation, will be presented in Section 8 “Discussion”.

## 8. Discussion

In the present work, the main objective was to provide a new simple analysis on the deformation process in tube drawing. Two approaches were applied, a FE simulation and an analytical calculation. In the analytical work, some of the major features found in the FE simulation were adopted. The principal finding in the FE simulation was the identification of the second main deformation zone as a near simple shear zone. This zone is situated at the intersection plane between the two corners of the deforming tube, where the material enters the fully constrained deformation zone, called Section IV of the processing. This finding was then the basis for the analytical modeling, which led to an analytical formula for the drawing force needed during the process Equation (48). The validity of this formula can be examined by comparing its prediction to the results of the FE calculations, as well as to experimental results. Table 3 shows the parameter values of the experiment that were entered into Equation (48). The obtained result is displayed in Table 4 as a function of the friction coefficient (the only unknown parameter of the process).

In Table 4, we have specified the drawing force, calculated with equal friction coefficients. This can be justified by the fact that the die, as well as the plug, were made from the same hard steel material and we used the same lubricant for both the die and the plug. Moreover, the compression stresses that control the friction force differed by only 5.7% according to Equation (36). A low dependence of the Coulomb friction parameter on the contact pressure is found in dry conditions in [31] for steel, which also justifies the use of the same friction coefficient at both the external and internal surfaces of the tube.

The results of the present two models can also be compared to the only known previous analytical formula, which was established by Geleji in 1967 [19]. The Geleji formula is based on a combination of an empty tube drawing and a plug drawing, where the plug drawing stage is analogous to rod drawing, with the difference that a frictional force is added at the plug surface. The formula is quite complex (a short description of the Geleji model is provided in Appendix A), and requires more parameters than the newly presented one—compare the parameter values between Table 3 and Table 5. The Geleji formula is the following:

*F* = *T*1 + (*T*2 × *T*3 × *T*4 + *T*5) (50)

where

$$T1=\frac{2\cdot K\cdot t_{0}}{R_{k}}+0.58\cdot B\cdot K\cdot \theta ,\;T2=\frac{N}{2}\cdot \left[k_{\mathrm{fA}}+k_{f1}+\frac{k_{\mathrm{fA}}+k_{f1}-\left(\frac{2\cdot K\cdot t_{0}}{R_{k}}+0.58\cdot B\cdot K\cdot \theta \right)}{B}\right]$$

$${T3=\left[1+\frac{B-M+\mu \left(\frac{B-N}{\sin\theta }+d_{1}\pi \cdot \frac{t_{0}-t_{1}}{\tan\theta }\right)}{2N}\right]}^{-1}\;T4=\left[B-N+\mu \cdot \left(\frac{B-N}{\sin\theta }+d_{1}\cdot \pi \cdot \frac{t_{0}-t_{1}}{\tan\theta }\right)\right]$$

$$T5=0.58\cdot \frac{k_{\mathrm{fA}}+k_{f1}}{2}\cdot N\cdot \theta ,\;$$

$$K=\frac{k_{f0}+k_{\mathrm{fA}}}{2},\;B=\left[\left(D_{A}^{2}-d_{1}^{2}\right)\cdot \frac{\pi }{4}\right],\;M=\left(D_{2}^{2}-d_{2}^{2}\right)\cdot \frac{\pi }{4},\;N=\left(D_{1}^{2}-d_{1}^{2}\right)\cdot \frac{\pi }{4}$$

The Geleji equation includes several forming strengths at different stages of drawing; they are the first five parameters in Table 5. In our new formula, only one forming strength is required, which is at the end of the tube drawing, expressed by the shear flow stress *τ*_0_. Its basic principles and the calculation process are presented in Appendix B.

**Figure 18:**
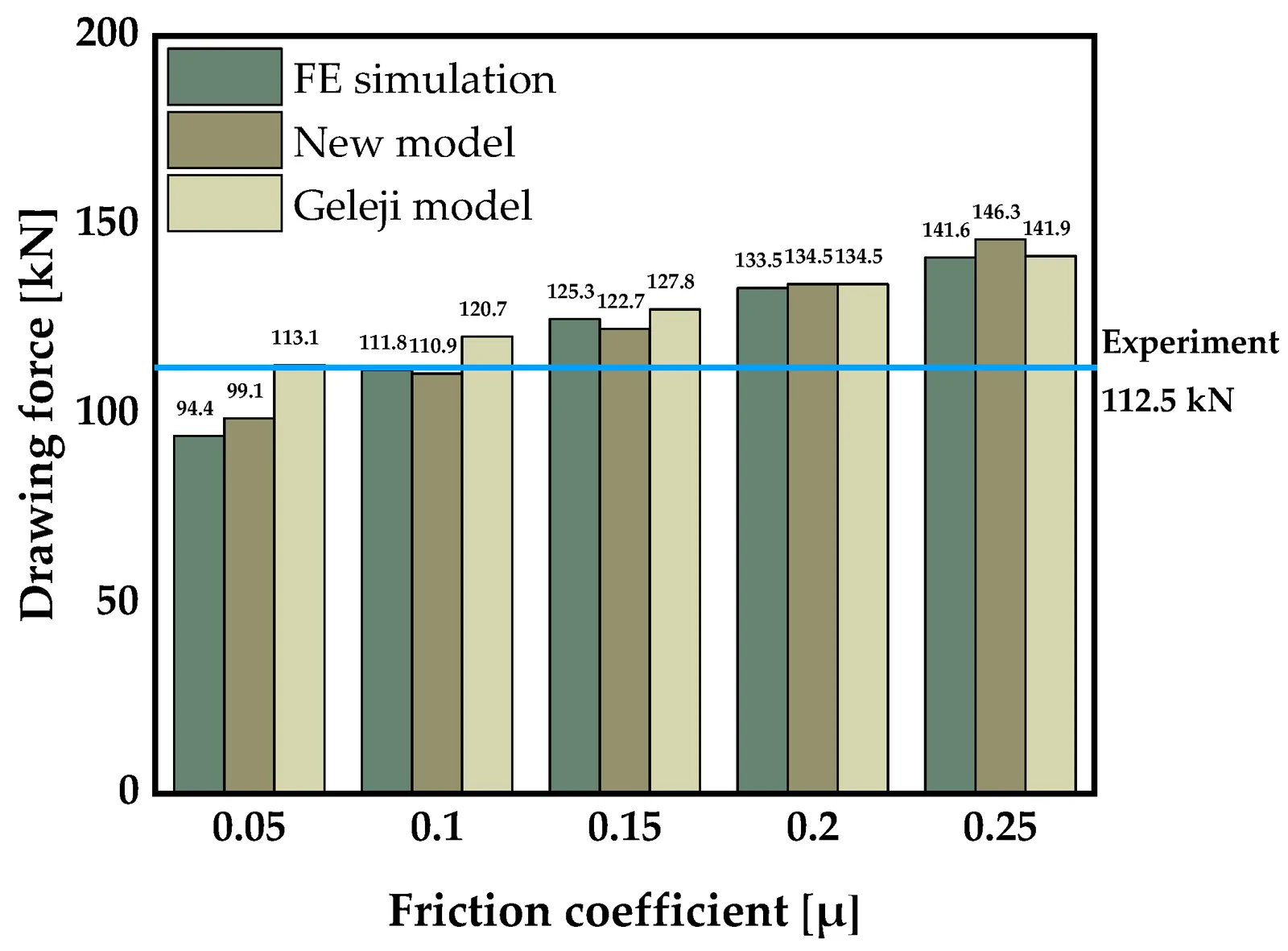
Analytical and FE simulated drawing forces in comparison with the experimental value.

**Figure 19:**
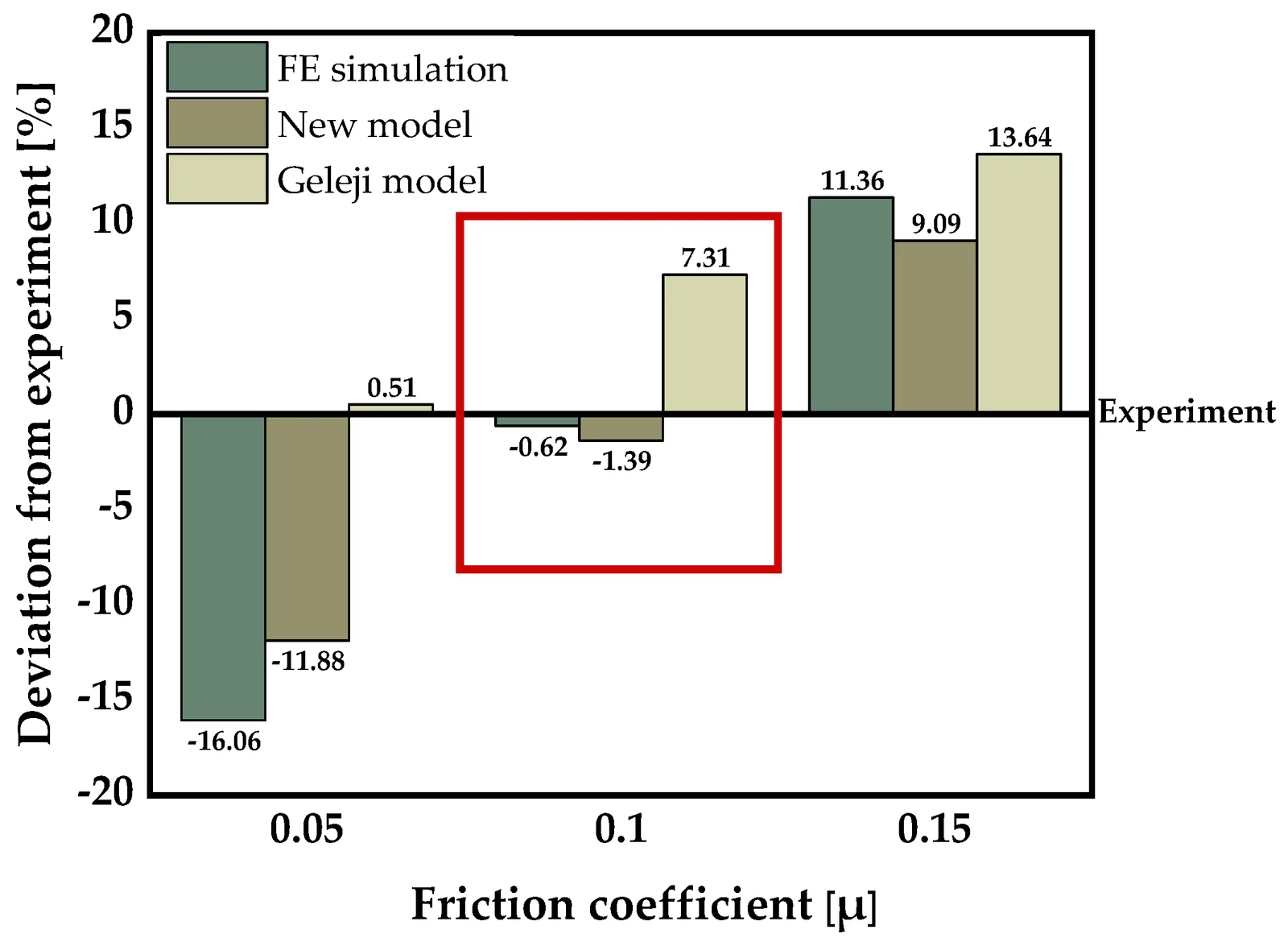
Magnitude of deviation of the drawing forces from the experimental value for the three models. The red box highlights the results corresponding for a friction coefficient of approximately 0.1, which corresponds to the actual friction condition of the drawing process.

Figure 18 shows the result obtained in form of a histogram for the three modelling approaches as a function of the friction coefficient. Figure 19 displays the differences with respect to the experimental value. Note that it is not common for the coefficient of friction to exceed 0.15 for cold tube drawing, due to evolution in the quality of the lubricants and the tooling materials. Therefore, the best agreements between the experimental drawing force and the predicted one are obtained for the presented analytical model and the FE simulation for *μ* = 0.1, with slightly better result for the analytical model. The Geleji model only performed well for very low friction (0.05).

The crystallographic texture was also analyzed in order to compare the performance of the simulation and the two analytical approaches. Note that for the Geleji formula it was not possible to carry out a texture simulation because the velocity gradients were not determined by Geleji. As shown in Section 7 “Texture Analysis”, both simulated textures agree well with the experimental one: 92% agreement achieved with the analytical model, and 91% by the FE simulation. Therefore, the velocity gradients of the two approaches and the obtained accumulated strains must have been similar. Concerning the equivalent strains in the analytical model; they were 0.193 and 0.276 in Sections III and IV, respectively, while the same were 0.189 and 0.271 in the FE simulation. So, the agreement is good.

The total strain in both approaches exceeds the last point on the experimental strain hardening curve by a strain of 0.23, so the extrapolation is not excessive. There is also an indirect validation of this extrapolation by the fact that the experimental texture produced was very close to the simulation by both approaches.

It is more complex to compare the velocity gradient tensors, because of the many components. It is done for Section IV in Figure 10, where the strain mode is constant simple shear according to the analytical model, while the velocity gradient components are varying in the FE case. The *L_rr_* and *L_zz_* components are equal with opposite signs, so the *L_θθ_* component is zero, like in the analytical model. The agreement between the two approaches is less good for the *L_rz_* and *L_zr_* components. In the analytical model, due to the near 45° inclination angle of the shear plane (*α* = 45.42°), these two components are almost equal but with opposite signs. That means a nearly perfect rigid body rotation around the *θ* axis, with a very small amount of plastic strain that corresponds to these velocity gradient components. This kind of velocity gradient was also observed in ECAP, see in Ref. [32]. However, in the FE simulation, these components have quite different values; their ratio is about −2, in contrast with the analytical approach, where it is near −1. One can calculate the value of the rigid body rotation rate corresponding to the two models. In the analytical model, according to Equation (14), it is:

(51) $$\dot{\omega }=\frac{L_{\mathrm{rz}}-L_{\mathrm{zr}}}{2}=-\dot{\left|\gamma \right|\cdot }\frac{\left({-\sin}^{2}\alpha -\cos^{2}\alpha \right)}{2}=\frac{\dot{\left|\gamma \right|}}{2}$$

The shear strain rate $\dot{\left|\gamma \right|}$ can be obtained from the value of the total shear in the shear zone (*γ* = 0.478) and the time spent there (*t* = 3.4 s):

(52) $$\dot{\left|\gamma \right|}=\gamma /t=0.478/3.4\;s=0.14\;s^{-1}$$

**Figure 20:**
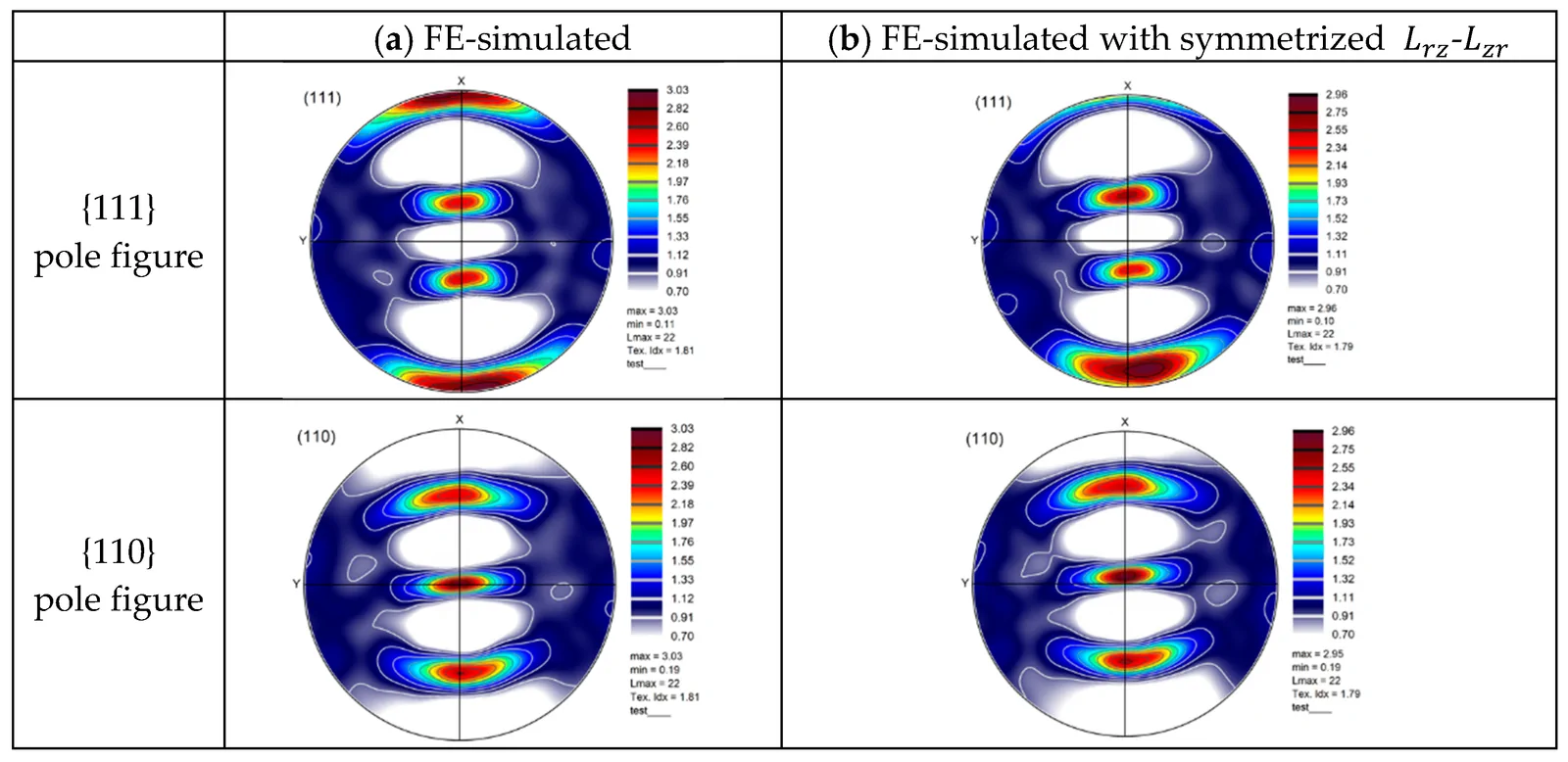
Simulated textures using the velocity gradient of the FE simulation only in the shear zone.

Considering that $\dot{\left|\gamma \right|}$ is constant in the simple shear model, we obtain from Equations (51) and (52) the angle of the rigid body rotation: ω = 13.6°. Similar calculations can be done in the FE case, however, for varying velocity gradient components. After integration, we obtain a rigid body rotation angle of also ~13.0°. It is very interesting that these values agree well with the experimentally observed EBSD pole figures, see Figure 17a. However, this texture rotation is only reproduced by the analytical model (Figure 17b). The FE-simulated pole figures are symmetric (Figure 17c). This is quite intriguing, because the same rigid body rotation is obtained from the integration of the *L_rz_* and *L_zr_* components of the FE simulation as in the simple shear model. In order to understand this peculiarity, special texture simulations were carried out for the FE simulation only in the “shear” section in section IV (Figure 10). The obtained textures are shown in Figure 20.

As can be seen in Figure 20a, the texture appears not rotated around the *y* (or *θ* axis), in spite of the presence of the *L_rz_* and *L_zr_* shear components in the FE-obtained velocity gradient tensor. Instead, it is slightly rotated around the *z* axis (the axis of the tube), which should be zero. From this non-admitted rotation, we can suspect that the precision of the FE simulation was not perfect. However, when the *L_rz_* and *L_zr_* components are artificially symmetrized, the pole figures are rotated by about +13° around the y axis, see Figure 20b. Then it follows from these two simulations, that the +13° rotation is cancelled by an opposite rotation! The origin of the opposite rotation actually is a plastic shear component which originates from the asymmetry of the *L_rz_* − *L_zr_* components. Indeed, there is a negative plastic shear value which corresponds to the imbalance of these shear components, which itself produces a negative rotation around the *y* axis, cancelling in this way the overall rotation of the texture. Nevertheless, large *L_rz_* and *L_zr_* components could be seen in the FE velocity gradient in Figure 10, in close similarity to the analytic simple shear model, together with large radial and axial components that also agree between the two approaches. Thus, the FE simulation confirms the existence of the shear zone but fails to predict the rotation around the *y* axis, simply because of insufficient accuracy in a FE simulation. The deviation from a fully precise simulation in the present FE calculation is also indicated by the production of another rotation of the texture (around the *z*-axis), which, however, is not admitted, because of the symmetry of the tube drawing process.

It is interesting to compare the obtained texture with the textures observed in cross-rolling of low carbon steel. In a recent paper by Toth et al. [33], such textures were examined, and one can see a striking similarity between the textures of tube-drawing and the textures of cross-rolling! However, there are three main differences between the two cases:

(i) In the observation planes in which the pole figures of the textures were observed: in cross rolling it is the plane of the sheet, while in tube drawing it is the plane perpendicular to the axis of the tube,
(ii) The plane of the plane strain state is changed in tube drawing, while in cross-rolling it is the rolling direction which is changed,
(iii) The {111} and {110} pole figures are exchanged in appearance.

To understand this, it is necessary to compare the velocity gradient tensors developed for Sections III and IV of the tube drawing. They are recalled here, for comparison:

(53) $$\underline{\underline{L}}\;\left(\mathrm{III}\right)={\left(\begin{array}{ccc} 0 & 0 & 0\\ 0 & -L_{\mathrm{zz}} & 0\\ 0 & 0 & L_{\mathrm{zz}} \end{array}\right)}_{r,\theta ,z}\;,\;\underline{\underline{L}}\;\left(\mathrm{IV}\right)=\dot{\frac{\left|\gamma \right|}{2}}{\left(\begin{array}{ccc} {-L}_{\mathrm{zz}} & 0 & L_{\mathrm{rz}}\\ 0 & 0 & 0\\ L_{\mathrm{zr}} & 0 & L_{\mathrm{zz}} \end{array}\right)}_{r,\theta ,z}$$

There is a rotation of 90° in the diagonal strain components, just like in cross rolling. However, it is the plane of plane strain which is rotated, not the rolling direction! That makes a radical difference in the strain mode compared to cross rolling, so it cannot be expected that the same pole figures are obtained; they are actually interchanged compared to cross rolling. One should not forget, nevertheless, about the shear component, which has the expression of *L_rz_* = (cos^2^*α* − sin^2^*α*) in Equation (15). However, the value of this component is relatively small with respect to the value of the normal strain rate component (sin2*α*), so the cross-type of deformation path is well approximated. At the same time, this relatively small shear component produces the small rotation of the texture around the tangential axis, as it is observed.

It is important to examine the limitations of the two presented models. The first limitation is the strain hardening extrapolation, which was needed because the strain in the simulation process exceeded the strain applied for the reference testing. Namely, when a tensile test is used to characterize the strain hardening behavior of the metal, it is always limited to relatively small strains because of the early strain localization in tensile testing. This can be improved by doing simple shear or torsion testing, where much larger strains can be reached. A second weak point is the issue of friction, which remains a tuning parameter in the presented models. This could be also improved by doing tribological tests on the same metal under the same compression force, temperature and speed as in the FPTD process.

Several interesting issues that can be important for further research in the FPTD process did not fit into the scope of the present paper due to length limitations. One is the strain gradient within the tube wall that can be examined by microstructure and texture analysis. It seems to be quite small in the present case, but could be more important in other geometries. Higher gradients can affect the precision of the simple shear approach. Another issue is the strain path change during FPTD. It was discovered in the present paper that there is a radical change in it when passing from Section III to Section IV of the deformation process. Such strain path change has a very strong influence on the texture that can be observed at the end of the deformation. The crystallographic texture is deterministic for the plastic anisotropy that can develop in the drawn tube.

## 9. Conclusions

In this paper, FE simulation was successfully applied to model the tube drawing process with a fixed plug. We also established a new analytical model for the stress and strain field from which the drawing force could be calculated. We found that the drawing force was predicted nearly equally well by the new model and the FE simulation, while the Geleji formula performed well only for low friction coefficient values. In order to verify the strain paths, the crystallographic texture was analyzed experimentally and also by using the velocity field obtained from the analytical model and from the simulation using the VPSC polycrystal model. From the results obtained, the following major conclusions can be drawn:

1. The new analytical formula provides nearly the same drawing force as the FE simulation. For reasonable friction coefficients, the best agreement with the measured drawing force was obtained by the new approach, while the FE simulation and the Geleji formula showed more deviations from the experimental value.
2. The crystallographic texture is a good (indirect) indicator of the modeled velocity gradient field in the viscoplastic self-consistent polycrystal approach. The simulated textures agreed 91% for FE simulation and 92% for the new analytical model.
3. There is an apparent high similarity of the crystallographic textures between the fixed-plug tube drawing and the texture in cross rolling, however, only if the corresponding pole figures are exchanged between {111} and {110}. The reason behind this is that in cross-rolling the rolling plane remains constant, while in tube drawing it is the axial direction is kept the same during the strain path changes. Another difference is that the plane of the strain mode is rotated by 90° during the passage of a material element from Section III to IV.

## Figures and Tables

**Table 1:** Chemical composition (wt%).

Sample ID	C	Mn	Si	P	S	Al
89117	0.14	0.50	0.02	0.012	0.013	0.037

**Table 2:** The parameter values of Equation (1) applied to the experimental tensile stress-strain curve.

*σ*_00_ [MPa]	*σ_sat_* [MPa]	*h* [MPa]	*a*	Average Deviation [MPa]
317	1201	48830	8.167	0.2

**Table 3:** Geometric parameter values used in the analytical formula to calculate the drawing force.

*τ*_0_ [MPa]	*θ* [rad]	*t*_0_ [mm]	*t*_1_ [mm]	*r*_1_ [mm]	*r*_2_ [mm]	*μ*
344.2	0.2323	3.0	2.5	17.5	15.0	0.05–0.25

**Table 4:** Predicted drawing forces obtained from FE simulation, from the analytical model, and from the Geleji formula. The experimental drawing force was 11.04 tons (=112.5 kN).

Friction Coefficient	FE Calculated Drawing Force [kN]	New Analytical Calculated Drawing Force [kN]	Deviation Between the New Model and FE [%]	Drawing Force from the Geleji Formula [kN]	Deviation Between Geleji and FE [%]
0.05	94.43	99.13	4.97	113.08	19.75
0.10	111.80	110.93	−0.77	120.73	7.98
0.15	125.29	122.73	−2.04	127.85	2.04
0.20	133.56	134.53	0.72	134.50	0.70
0.25	141.61	146.33	3.33	141.92	0.21

**Table 5:** Parameter values in the Geleji formula necessary to calculate the drawing force.

*k_f_*_0_ [MPa]	*k_fA_* [MPa]	*k_f_*_1_ [MPa]	*R_k_* [MPa]	*D_A_* [mm]	*D*_2_ [mm]	*D*_1_ [mm]	*d*_2_ [mm]	*d*_1_ [mm]	*t*_0_ [mm]	*t*_1_ [mm]	*θ* [rad]	*μ*
320	560	596.2	19.5	36	42	36	35	30	3.0	2.5	0.2323	0.05–0.25

**Figure A1:**
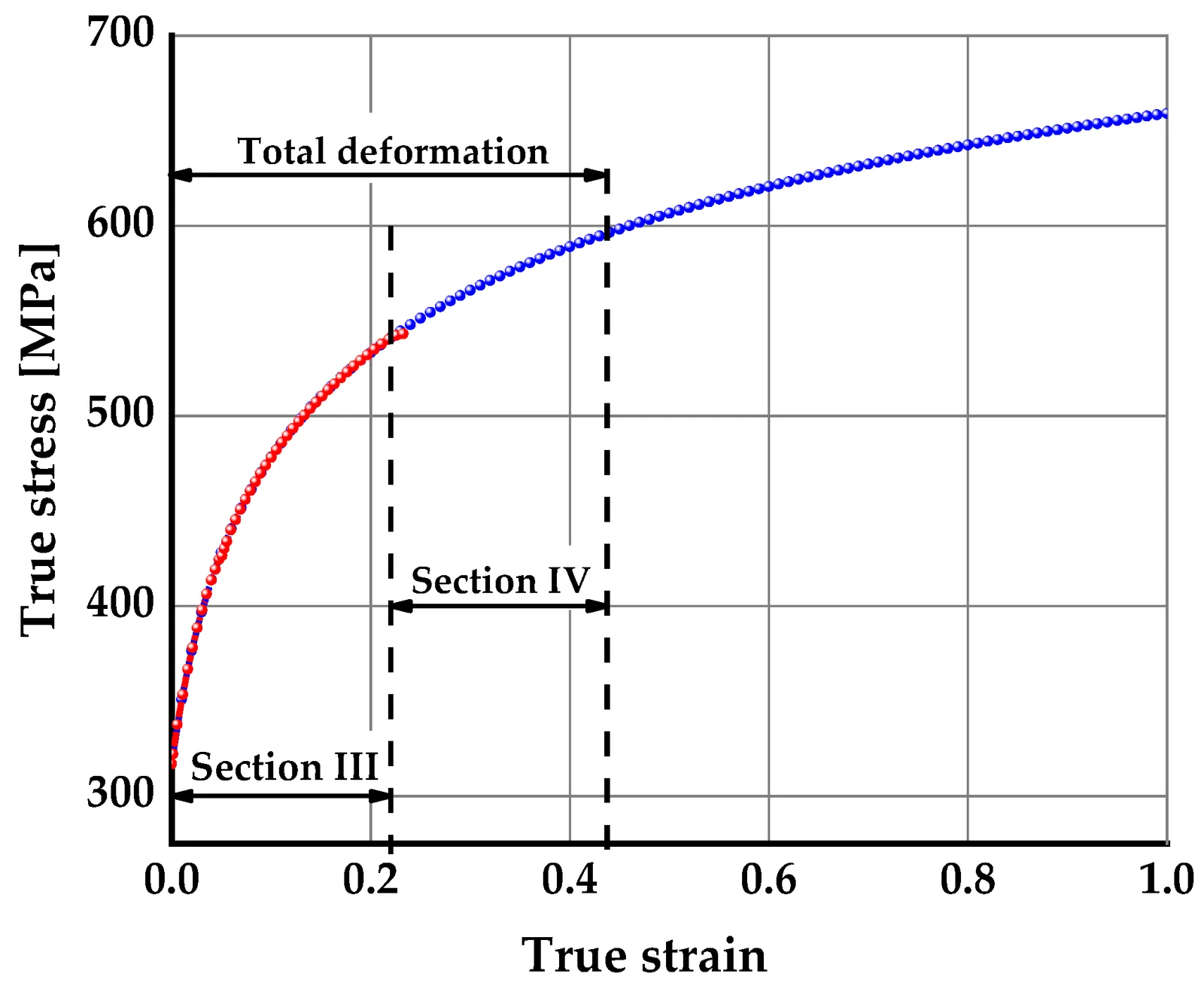
Experimental true stress–true strain curve (red line) in tensile testing and the fitted function (blue points).

## Data Availability

The original contributions presented in this study are included in the article. Further inquiries can be directed to the corresponding author.

## Appendix A

### A Short Description of the Geleji Model

Geleji divided the tube drawing process into two stages when determining the drawing force in fixed-plug tube drawing. He first considered Stage III, where the tube enters the drawing die, as a tube drawing without a plug, in which only the outer wall of the tube is in contact with the drawing die. In Stage IV, the outer surface of the tube is in contact with the drawing die and the inner surface is in contact with the fixed plug, analogous to bar drawing, since frictional force is generated also on the surface of the plug. The model is using an energy-based mechanical approach, in which the drawing force is determined as the sum of the contributions arising from the deformation resistance in the different sections of the die and from the friction between the die and the tube. In the calculation, the deformation resistance and the flow stress are assumed to be constant within each section of the die. (Note: The geometric dimensions and notations here are the same as those shown in Figure 2.)

The Geleji equation for determining the drawing force is as follows:

*F* = *F*_*III*_ + *F*_*IV*_ (A1)

The drawing force to be exerted in Stage III is:

(A2) $$F_{\;\mathrm{III}}=p_{\mathrm{kIII}}\cdot S_{I}^{*}\cdot \left(1+\frac{\mu }{\alpha }\right)+0.58k_{\mathrm{fkIII}}\cdot S_{A}\alpha$$

Here, *p_kIII_* is the specific contact pressure in Stage III:

(A3) $$p_{\mathrm{kIII}}=\frac{k_{\mathrm{fkIII}\;}}{\frac{R_{k}}{t_{0}}\cdot \frac{S_{\mathrm{III}}^{*}}{2S_{A}}\cdot \left(1+\frac{\mu }{\alpha }\right)}$$

Here $S_{\mathrm{III}}^{*}$ is the “apparent” reduction in Stage III:

(A4) $$S_{\mathrm{III}}^{*}=\left(D_{2}^{2}-D_{A}^{2}\right)\cdot \frac{\pi }{4}$$

The average forming strength in Stage III:

(A5) $$k_{\mathrm{fkIII}}=\frac{k_{f0}+k_{\mathrm{fA}}}{2}$$

The drawing force to be exerted in Stage IV is:

*F_IV_* = *k_kIV_* ∙ [*S_IV_* + *μ* ∙ (*Q_szIV_* + *Q_dIV_*)] + 0.58 ∙ *k_fkIV_* ∙ *S*_1_ ∙ *α* (A6)

The average forming strength in Stage IV, considering the *F_III_* pullback force:

(A7) $$k_{\mathrm{kIV}}=\frac{k_{\mathrm{fkIV}}-\frac{F_{\;\mathrm{III}}}{2S_{A}}}{1+\frac{S_{\mathrm{IV}}+\mu \left(Q_{\mathrm{szIV}}+Q_{\mathrm{dIV}}\right)}{2S_{1}}}$$

where the working surface of the die is:

(A8) $$Q_{\mathrm{szIV}}=\frac{S_{\mathrm{IV}}}{\sin\alpha }$$

where the actual reduction in Stage IV:

*S_IV_* = *S_A_* − *S*_1_ (A9)

and the working surfaces of the plug:

(A10) $$Q_{\mathrm{dIV}}=d_{1}\pi \cdot \frac{t_{0}-t_{1}}{\tan\alpha }$$

The average forming strength in Stage IV is:

(A11) $$k_{\mathrm{fkIV}}=\frac{k_{\mathrm{fA}}+k_{f1}}{2}$$

Finally, the fully substituted Geleji formula is as follows:

(A12) $$F=\left[\frac{\frac{k_{f0}+k_{\mathrm{fA}}}{2}}{\frac{R_{k}}{t_{0}}\cdot \left[\frac{\left(D_{2}^{2}-D_{A}^{2}\right)\cdot \frac{\pi }{4}}{2\left(D_{A}^{2}-d_{1}^{2}\right)\cdot \frac{\pi }{4}}\right]\cdot \left(1+\frac{\mu }{\alpha }\right)}\cdot \left[\left(D_{2}^{2}-D_{A}^{2}\right)\cdot \frac{\pi }{4}\right]\cdot \left(1+\frac{\mu }{\alpha }\right)+0.58\frac{k_{f0}+k_{\mathrm{fA}}}{2}\cdot \left[\left(D_{A}^{2}-d_{1}^{2}\right)\cdot \frac{\pi }{4}\right]\cdot \alpha \right]+\;\;\frac{\frac{k_{\mathrm{fA}}+k_{f1}}{2}-\frac{\left[\frac{\frac{k_{f0}+k_{\mathrm{fA}}}{2}}{\frac{R_{k}}{t_{0}}\cdot \left[\frac{\left(D_{2}^{2}-D_{A}^{2}\right)\cdot \frac{\pi }{4}}{2\left(D_{A}^{2}-d_{1}^{2}\right)\cdot \frac{\pi }{4}}\right]\cdot \left(1+\frac{\mu }{\alpha }\right)}\cdot \left[\left(D_{2}^{2}-D_{A}^{2}\right)\cdot \frac{\pi }{4}\right]\cdot \left(1+\frac{\mu }{\alpha }\right)+0.58\frac{k_{f0}+k_{\mathrm{fA}}}{2}\cdot \left[\left(D_{A}^{2}-d_{1}^{2}\right)\cdot \frac{\pi }{4}\right]\cdot \alpha \right]}{2\left[\left(D_{A}^{2}-d_{1}^{2}\right)\cdot \frac{\pi }{4}\right]}}{1+\frac{\left[\left(D_{A}^{2}-d_{1}^{2}\right)\cdot \frac{\pi }{4}-\left(D_{2}^{2}-d_{2}^{2}\right)\cdot \frac{\pi }{4}\right]+\mu \left(\frac{\left(D_{A}^{2}-d_{1}^{2}\right)\cdot \frac{\pi }{4}-\left(D_{1}^{2}-d_{1}^{2}\right)\cdot \frac{\pi }{4}}{\sin\alpha }+d_{1}\pi \cdot \frac{t_{0}-t_{1}}{\tan\alpha }\right)}{2\cdot \left[\left(D_{1}^{2}-d_{1}^{2}\right)\cdot \frac{\pi }{4}\right]}}\;\;\left.\left[\left[\left(D_{A}^{2}-d_{1}^{2}\right)\cdot \frac{\pi }{4}-\left(D_{1}^{2}-d_{1}^{2}\right)\cdot \frac{\pi }{4}\right]+\mu \cdot \left(\frac{\left(D_{A}^{2}-d_{1}^{2}\right)\cdot \frac{\pi }{4}-\left(D_{1}^{2}-d_{1}^{2}\right)\cdot \frac{\pi }{4}}{\sin\alpha }+d_{1}\pi \cdot \frac{t_{0}-t_{1}}{\tan\alpha }\right)\right]+0.58\cdot \frac{k_{\mathrm{fA}}+k_{f1}}{2}\cdot \left[\left(D_{1}^{2}-d_{1}^{2}\right)\cdot \frac{\pi }{4}\right]\cdot \alpha \right]$$

## Appendix B

### Calculation of the Material Strength at the Exit Point in Tube Drawing

In order to determine the value of *τ*_0_, we need first to determine the total deformation during the tube drawing process, accumulated in Sections III and IV. Equation (9) provides the accumulated strain in Section III as a function of the radial exit position. As the dependence on the radial position is quite small (about 2% within the thickness of the tube), the average value between the internal and external surfaces of the tube was calculated by:

(A13) $${\bar{\varepsilon }}_{\mathrm{III}}=\frac{1}{\sqrt{3}}\ln\left(\frac{R_{1}}{r_{1}}+\frac{R_{2}}{r_{2}}\right)$$

The strain field is homogeneous in the shear zone, so the equivalent von Mises strain can be calculated by the following formulas:

(A14) $$\varepsilon_{\mathrm{IV}}=\frac{\gamma }{\surd 3},\;\mathrm{where}\;\gamma =\cot\alpha +\cot\beta$$

By adding the deformations of the two stages, we obtain the total deformation:

(A15) $$\varepsilon =\frac{1}{\sqrt{3}}\left[\ln\left(\frac{R_{1}}{r_{1}}+\frac{R_{2}}{r_{2}}\right)+\cot\alpha +\cot\beta \right]$$

The deformation strength value corresponding to the total deformation value can be read from the strain hardening function curve for the given material (Figure A1), or simply by substituting the total deformation value into our strain hardening function Equation (1).

The shear strength can then be obtained from the tensile strength *k_f_*, corresponding to the true strain *ε* by:

(A16) $$\tau_{0}\left(\varepsilon \right)=\frac{k_{f}\left(\varepsilon \right)}{\sqrt{3}}$$

## References

[1] Kuboki T., Tasaka S., Kajikawa S. (2017). Examination of working condition for reducing thickness variation in tube drawing with plug. COMPLAS XIV, Proceedings of the XIV International Conference on Computational Plasticity: Fundamentals and Ap-plications, Barcelona, Spain, 5–7 September 2017.

[2] Zhanga L., Xua W., Longa J., Lei Z. (2015). Surface roughening analysis of cold drawn tube based on macro–micro coupling finite element method. J. Mater. Process. Technol..

[3] Luksza J., Burdek M. (2002). The influence of the deformation mode on the final mechanical properties of products in multi-pass drawing and flat rolling. J. Mater. Process. Technol..

[4] Dieter G.E., Kuhn H.A., Semiatin S.L. (2003). Chapter 19 Drawing of Wire, Rod, and Tube. Handbook of Workability and Process Design.

[5] Bella P., Bucek P., Ridzon M., Mojzis M., Parilak L. (2017). On numerical modelling of multi-rifled tube drawing. Procedia Eng..

[6] Al-Hamdany N., Salih M.Z., Palkowski H., Carradò A., Gan W., Schell N., Brokmeier H.-G. (2021). Tube Drawing with Tilted Die: Texture, Dislocation Density and Mechanical Properties. Metals.

[7] Razali N.A., Byun J.B., Joun M.S. (2024). Novel finite element model of analyzing wall thickness during tube drawing considering raw tube’s thickness non-uniformity and die misalignment. Int. J. Mater. Form..

[8] Gattmah J., Ozturk F., Orhan S. (2020). A new development of measurement technique for residual stresses generated by the cold tube drawing process with a fixed mandrel. Int. J. Adv. Manuf. Technol..

[9] Suliga M., Mróz S., Szota P., Wasilewski M., Jaroszewski K. (2025). Numerical Analysis of the Process of Drawing Thin-Walled CuSn8 Alloy Tubes on a Cylindrical Plug. Materials.

[10] Khani S., Palkowski H., Carradò A., Foadian F. (2023). Experimental Investigation on Local and Global Texture Evolution in Drawing Seamless Copper Tubes. Appl. Mech..

[11] Avitzur B. (1983). Chapter 9 Tubing and Tubular products. Handbook of MetalForming Process.

[12] Neves F.O., Button S.T., Caminaga C., Gentile F.C. (2005). Numerical and Experimental Analysis of Tube Drawing with Fixed Plug. J. Braz. Soc. Mech. Sci. Eng..

[13] Um K.K., Lee D.N. (1997). An upper bound solution of tube drawing. J. Mater. Process. Technol..

[14] Rubio E.M., González C., Marcos M., Sebastián M.A. (2006). Energetic analysis of tube drawing processes with fixed plug by upper bound method. J. Mater. Process. Technol..

[15] Zhao D.W., Du H.J., Wang G.J., Liu X.H., Wang G.D. (2009). An analytical solution for tube sinking by strain rate vector inner-product integration. J. Mater. Process. Technol..

[16] Rubio E.M. (2006). Analytical methods application to the study of tube drawing processes with fixed conical inner plug: Slab and Upper Bound Methods. J. Achiev. Mater. Manuf. Eng..

[17] Tisza M. (1996). Metal Forming.

[18] Szombathelyi V., Krállics G. (2013). Csőhúzási Folyamatok Modellezése az Energetikai Módszer Alapján [Modelling of Tube Drawing Processes Based on the Energy Method].

[19] Geleji S. (1967). A Fémek Képlékeny Alakításának Elmélete [The Theory of Plastic Deformation of Metals].

[20] Siebel E. (1932). Die Formgebung im Bildsamen Zustande [Plastic Deformation of Metals].

[21] Yoshida K., Furuya H. (2004). Mandrel drawing and plug drawing of shape-memory-alloy fine tubes used in catheters and stents. J. Mater. Process. Technol..

[22] (2019). Metallic Materials — Tensile Testing — Part 1: Method of Test at Room Temperature.

[23] Beausir B., Fundenberger J.-J. (2017). ATEX-Software.

[24] Sahoo S.K., Dhinwal S.S., Vu V.Q., Toth L.S. (2021). A new macroscopic strain hardening function based on microscale crystal plasticity and its application in polycrystal modeling. Mater. Sci. Eng..

[25] MSC Marc Software.

[26] Hasani A., Tóth L.S., Beausir B. (2010). Principles of Nonequal Channel Angular Pressing. J. Eng. Mater. Technol..

[27] Lee D.N. (2000). An Upper-Bound Solution of Channel Angular Deformation. Scr. Mater..

[28] Lebensohn R.A., Tome C.N. (1993). A self-consistent anisotropic approach for the simulation of plastic deformation and texture development of polycrystals: Application to zirconium alloys. Acta Metall. Mater..

[29] Kalidindi S.R., Bronkhorst C.A., Anand L. (1992). Crystallographic texture evolution in bulk deformation processing of FCC metals. J. Mech. Phys. Solids.

[30] Molinari A., Toth L.S. (1994). Tuning A Self-Consistent Viscoplastic Model by Finite-Element Results-I. Modeling. Acta Metall. Mater..

[31] Pougis A., Philippon S., Massion R., Faure L., Fundenberger J.-J., Toth L. (2013). S: Dry friction of steel under high pressure in quasi-static conditions. Tribol. Int..

[32] Tóth L.S., Massion R.A., Germain L., Baik S.C., Suwas S. (2004). Analysis of texture evolution in equal channel angular extrusion of copper using a new flow field. Acta Mater..

[33] Toth L.S., Marouani J., Szűcs M., Amininejad A. (2026). Cross-rolling of low-carbon steel: Experiments and high-fidelity polycrystal modeling for texture evolution. J. Mater. Sci. Met. Corros..

